# A quantitative systems pharmacology approach, incorporating a novel liver model, for predicting pharmacokinetic drug-drug interactions

**DOI:** 10.1371/journal.pone.0183794

**Published:** 2017-09-14

**Authors:** Mohammed H. Cherkaoui-Rbati, Stuart W. Paine, Peter Littlewood, Cyril Rauch

**Affiliations:** 1 School of Veterinary Medicine and Science, University of Nottingham, Sutton Bonington, Leicestershire, United Kingdom; 2 Vertex Pharmaceuticals (Europe) Limited, Abingdon, Oxfordshire, United Kingdom; National Chiao Tung University College of Biological Science and Technology, TAIWAN

## Abstract

All pharmaceutical companies are required to assess pharmacokinetic drug-drug interactions (DDIs) of new chemical entities (NCEs) and mathematical prediction helps to select the best NCE candidate with regard to adverse effects resulting from a DDI before any costly clinical studies. Most current models assume that the liver is a homogeneous organ where the majority of the metabolism occurs. However, the circulatory system of the liver has a complex hierarchical geometry which distributes xenobiotics throughout the organ. Nevertheless, the lobule (liver unit), located at the end of each branch, is composed of many sinusoids where the blood flow can vary and therefore creates heterogeneity (*e*.*g*. drug concentration, enzyme level). A liver model was constructed by describing the geometry of a lobule, where the blood velocity increases toward the central vein, and by modeling the exchange mechanisms between the blood and hepatocytes. Moreover, the three major DDI mechanisms of metabolic enzymes; competitive inhibition, mechanism based inhibition and induction, were accounted for with an undefined number of drugs and/or enzymes. The liver model was incorporated into a physiological-based pharmacokinetic (PBPK) model and simulations produced, that in turn were compared to ten clinical results. The liver model generated a hierarchy of 5 sinusoidal levels and estimated a blood volume of 283 mL and a cell density of 193 × 10^6^ cells/g in the liver. The overall PBPK model predicted the pharmacokinetics of midazolam and the magnitude of the clinical DDI with perpetrator drug(s) including spatial and temporal enzyme levels changes. The model presented herein may reduce costs and the use of laboratory animals and give the opportunity to explore different clinical scenarios, which reduce the risk of adverse events, prior to costly human clinical studies.

## Introduction

A pharmacokinetic drug-drug interaction (DDI) is where a drug(s), the perpetrator drug(s), interacts with a metabolizing enzyme(s) or membrane transporter(s) such that the pharmacokinetics (PK) of another drug(s), the victim drug(s), is altered. In the late 1970s, the first cases of pharmacokinetic DDIs were reported [1], and since then more and more DDIs have been identified especially in the situation of polypharmacy as is often the case for elderly patients [2]. The increase in observed DDIs coupled to some lethal cases [1] led the FDA to publish in 1997 the first *in vitro* metabolism drug interaction guidance document [3] for pharmaceutical companies. In order to identify the possible interactions of new chemical entities (NCEs), many strategies have been suggested as it is essential to know before costly clinical trials, whether a NCE will be a safe drug. One of those strategies relies on the combination of *in vitro* information coupled to mathematical models to predict the clinical DDIs. This has the advantage to be cost effective, reduce the use of laboratory animals and give the opportunity to explore different clinical scenarios in order to identify optimum dose regimens. Excluding the limitations of *in vitro* experiments, the modeling approach is limited by the sophistication of the implemented models. Current models are classified into two different categories that depend on whether they are a function of time or not (*i*.*e*. static and dynamic models), and mainly focus on one enzyme and/or one particular aspect of DDIs (*e*.*g*. reversible inhibition [4], mechanism based inhibition [5] or induction [6]). In their most advanced form the static models may account for all kinds of DDIs [7], but are limited in their ability to describe complex mechanisms related to administration, distribution, metabolism or excretion such as active drug transport (uptake) into hepatocytes or enterocytes. Although the dynamic models are more descriptive, traditionally, the dynamic models were developed to describe specific drug cases [4, 5, 8] and most of them assume that the liver is a homogeneous organ (*e*.*g*. well-stirred model [9, 10]) where the majority of the metabolism occurs. However, the circulatory system of the liver has a complex hierarchical geometry which helps to distribute xenobiotics throughout the organ. Nevertheless, the lobule (liver unit), located at the end of each branch, is composed of many sinusoids (small blood vessels) where the blood flow can vary and therefore creates heterogeneity (*e*.*g*. drug concentration, enzyme level). Some liver models account for heterogeneity, such as the parallel tube model [9, 10] and the dispersion model [11, 12], but they have not been used to predict DDIs and do not account for the variation in blood flow through the lobules. With established methodologies of *in vitro* screening for DDIs, pharmaceutical companies need adequate tools to predict the net result of *in vivo* DDIs to translate their *in vitro* observations to clinical predictions. It is common for elderly patients to receive several medications to treat different symptoms or conditions. Each of these medicines can potentially interfere with the usual routes of metabolism for another drug. There is a serious need for better models to cover all different scenarios, which also takes into account the variabilities between individuals, such as size, weight and differences in genetic polymorphisms [13]. In this paper, a liver model will be presented that takes into account three major DDI mechanisms of metabolic enzymes; competitive inhibition, mechanism based inhibition (MBI) and induction, with an undefined number of drugs and/or enzymes, where the lobule geometry will be accounted for due to its impact on blood flow heterogeneity. The liver model will then be incorporated into a physiological-based pharmacokinetic (PBPK) model and simulations produced that in turn will be compared to clinical results. The description of the model proposed herein is divided into six parts. The first part will introduce the model and the notations used throughout the document including a new liver model taking into consideration its hierarchical structure and the different body compartments that are essential to drug metabolism. In the second part, the algorithm to generate the lobule geometry will be presented, where length and radius of the sinusoids are produced. In the third part, the transport and metabolism reactions of the drugs will be mathematically described. As the drugs are distributed in the body through the bloodstream, the conservation equation will be used in the liver sinusoids to describe the blood transport and the exchange mechanisms between the blood and hepatocytes, such as passive diffusion and active uptake/efflux of the drugs. Inside the hepatocytes, drug metabolism and drug interactions with metabolic enzymes will be described. In the fourth part, the PBPK model presented in part one will be fully developed. In part five, a brief description on how the PBPK was numerically resolved will be given. In the sixth part, drugs for which data exist will be considered and their physiological parameters defined. Finally the results from the new liver model will be presented and compared to clinical data.

## Models

### Presentation of the liver model and notations

The objective of this section is to provide a brief explanation of the subsequent models that will be used to develop a formal understanding of DDIs. There are three major aspects to consider; (i) the geometry of the lobule (ii) the set of complex interactions between xenobiotics and enzymes (iii) the usual set of body compartments (PBPK Model).

**Fig 1:**
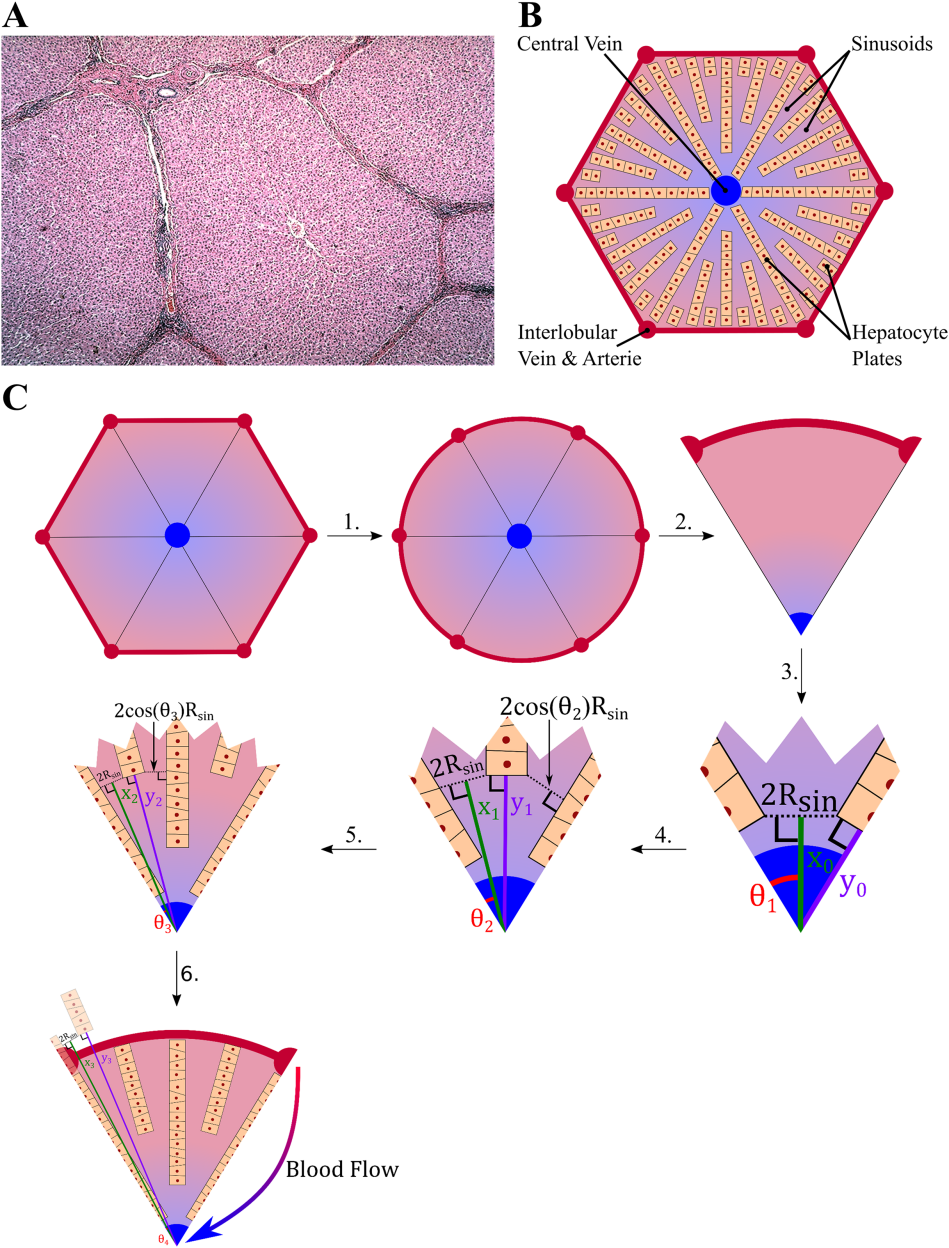
Lobule geometry and modelling. (A) The lobule cross section as represented displays an apparent elementary symmetry essential for its physiology primarily given by the blood vessels and the blood flow (Credit to Dr. Roger C. Wagner, University of Delaware). (B) This symmetry is used when lobule modeling or representation are involved. In general, a lobule is represented by a hexagon composed of hepatocyte plates. These plates are hierarchically organized to optimize exchanges. (C) To model the blood flow (and subsequent exchanges between the liver tissues and the blood), an algorithm was designed to automatically generate the length and radius of the sinusoids. The latter is used to estimate the changes in velocity within a sinusoid portion by assuming a constant blood flow and a constant velocity over the cross section.

Drugs move with the flow of blood and as a result the exchange mechanisms of drug between the blood and the tissue will be a function of the lobule geometry in which the flow takes place. The lobules have a peculiar shape idealized as hexagons composed of a series of peripheral entries (portal veins and hepatic arteries) and a central vein (Fig 1A and 1B). This spatial configuration and its hierarchical structure will need to be taken into consideration in order to describe the blood flow and to generate an algorithm to construct a lobule within the physiological constraints. Furthermore, the blood velocity will be assumed constant and averaged over the cross section of sinusoids present within the lobule while it will vary along the length of sinusoids as their radii narrow. The latter assumption is the only one that will be used, which reduces the spatial dimension to one. The spatial variable is noted *x* and for each sinusoids portion the *x*-axis is taken along the bisector and goes from the external part of the lobule to the central vein (Fig 1C).

To describe the DDIs no assumptions will be made on the number of drugs and enzymes involved to make the model generic for all animal species. However, the only way to achieve this is to consider matrix calculus. While this may appear as an unnecessary complication at first sight, it will be seen that with adequately defined operators the writing of equations is largely intuitive even for those not fully familiar with matrix algebra. To start with, the following notations *n*_*C*_ and *n*_*E*_ will refer to the number of drugs and enzymes, respectively. To distinguish between scalars and matrices (including vectors), matrices and vectors are written in bold. Thus any set of variables or constants related to drugs shall be described as a column vector of size *n*_*C*_ including their concentrations: ***C*** = (*C*_1_⋯*C*_*n*_*C*__)^*tr*^ or membrane permeability: ***P*** = (*P*_1_⋯*P*_*n*_*C*__)^*tr*^. Note here that the subscript “*tr*” refers to the transposition of a column vector into a vector line (the same notation shall be used for matrices where in this case the operator transposed is noted: $A^{tr}={\left(a_{i,j}^{tr}\right)}_{\begin{array}{c} 1\leq i\leq m\\ 1\leq j\leq n \end{array}}={\left(a_{j,i}\right)}_{\begin{array}{c} 1\leq i\leq m\\ 1\leq j\leq n \end{array}}$ where $A={\left(a_{i,j}\right)}_{\begin{array}{c} 1\leq i\leq n\\ 1\leq j\leq m \end{array}}$). Similarly, any set of variables or constants related to enzymes or their degradation shall be described as a column vector of size *n*_*E*_ including for example the enzyme concentrations: ***E*** = (*E*_1_⋯*E*_*n*_*E*__)^*tr*^; or their degradation: ***k***_***deg***_ = (*k*_*deg*, 1_⋯*k*_*deg*, *n*_*E*__)^*tr*^. As the number of possible pair interactions between enzymes and drugs is given by the scalar product: *n*_*C*_ × *n*_*E*_, one defines the matrix $EC={\left(EC_{i,j}\right)}_{\begin{array}{c} 1\leq i\leq n_{C}\\ 1\leq j\leq n_{E} \end{array}}$ where the term *EC*_*i*, *j*_ represents the interaction of the *i*-th drug with the *j*-th enzyme. Finally, as the interaction between the *i*-th drug with the *j*-th enzyme can lead to the formation of a product one needs to specify the kinetics of the reaction by another parameter, *k*_*cat*, *i*, *j*_, specific to the *EC*_*i*, *j*_ complex. In these conditions the scalar product *k*_*cat*, *i*, *j*_ × *EC*_*i*, *j*_ define the reaction rate of the reaction. To use matrices one needs to define the following operators: “⋅” such that $k_{cat}\cdot EC={\left(k_{cat,i,j}\times EC_{i,j}\right)}_{\begin{array}{c} 1\leq i\leq n_{C}\\ 1\leq j\leq n_{E} \end{array}}$. By extension, a division operator is defined and noted “/” or “−” between vectors or matrices such that: $x/y=\frac{x}{y}={\left(\frac{x_{i,j}}{y_{i,j}}\right)}_{\begin{array}{c} 1\leq i\leq n\\ 1\leq j\leq m \end{array}}$. Finally for completion the column vectors of size *n*_*C*_ or *n*_*E*_ and of components equal to unity shall be noted: $1_{n_{C}}$ and $1_{n_{E}}$.

**Fig 2:**
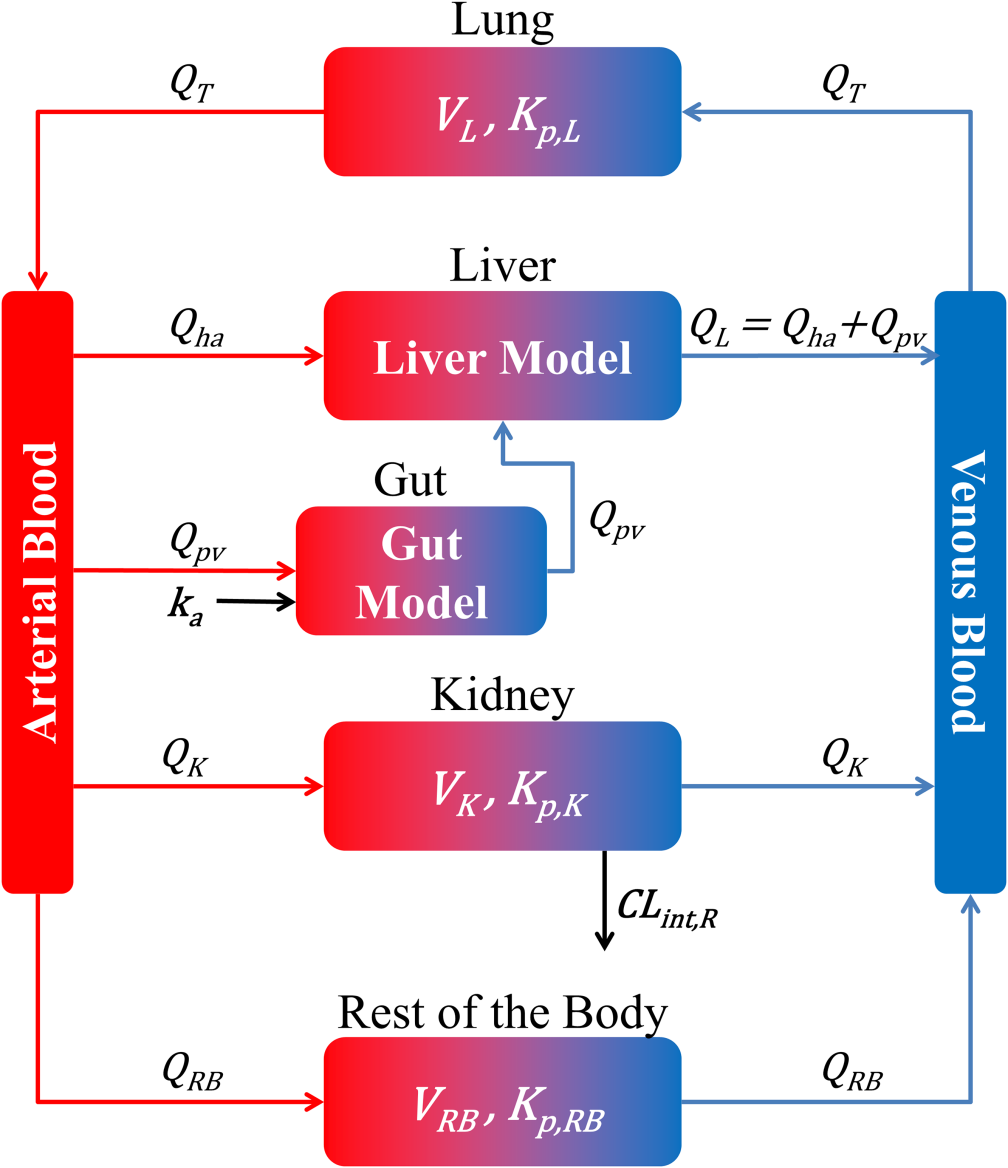
The seven compartmental model. Red and blue arrows represent blood flows (*Q*_*i*_ where *i* represents: *T* for total blood flow, *ha* hepatic artery blood flow, *pv* portal vein blood flow, *L* for the liver blood flow, *G* for the gut blood flow, *K* for the kidneys blood flow and *RB* for the blood flow going to the rest of the body). The black arrows represent absorption (*k*_*a*_: absorption constant rate) or excretion (*CL*_*R*_: Renal Clearance).

Last but not least, a seven-compartment model is used involving: venous blood, arterial blood, liver, gut, kidneys, lungs (to consider the pulmonary circulation), and the rest of the body (Fig 2). The average volume and blood flow of each compartment is given in S1 Table.

### Lobule geometry

The lobule is the elementary unit of the liver where the exchange of nutrients and xenobiotic compounds occurs between the blood and the hepatocytes. The shape of the lobule and the spatial distribution of the hepatocytes are irregular in appearance (Fig 1A). But schematically, the liver lobule can be represented by a hexagon (Fig 1B), where the hepatic vein is at the centre and where at each apex, the hepatic artery and the hepatic portal vein pour blood into the sinusoids. The sinusoids are converging toward the centre where the blood leaves the lobule through the central vein (Fig 1B).

To suggest a theory on which an amenable model will be based, the lobule geometry will be simplified. The parameters used to define the geometry are summarized in Table 1, whereas the algorithm to build the lobule geometry for the simulations is schematically represented in Fig 1C where each step is numbered and detailed as below:

1. The hexagonal shape of the lobule is replaced by a disc of similar area: $\frac{3\sqrt{3}}{2}{\bar{R}}_{lobule}^{2}=\pi R_{Circle}^{2}$.
2. Due to the symmetry of an hexagon, only one sixth of the circle will be taken into account.
3. The initial two hepatocyte plates are placed on either edge of the sector (one sixth of the circle) and respect a minimal distance of 2*R*_*Sin*_ between the hepatocytes plates, the initial distances from the centre are estimated by:
  - The first sinusoid output: $x_{0}=\frac{R_{Sin}}{\tan\theta_{1}}+\frac{e}{2\sin\theta_{1}}$ where $\theta_{k}=\frac{\theta_{0}}{2^{k}}$ and *θ*_0_ = 60°.
  - The first hepatocyte plate: $y_{0}=\frac{R_{Sin}}{\sin\theta_{1}}+\frac{e}{2\tan\theta_{1}}$.
  - Iteration initialization: *k* = 0
4. A loop is implemented as follow: while *x*_*k*_ ≤ *R*_*Circle*_ do
  - *k* = *k* + 1
  - Place a hepatocyte plate on each line of angle *θ*_*k*_ + (*i* − 1)*θ*_*k* − 1_ for ∀*i* ∈ {1, …, 2^*k* − 1^}, such as the minimal distance of the new hepatocyte plate to the previous one is 2*R*_*Sin*_ cos *θ*_*k* + 1_, which gives an output diameter of 2*R*_*Sin*_.
  - The distance of the outputs: $x_{k}=\frac{R_{Sin}}{\tan\theta_{k+1}}+\frac{e}{2\sin\theta_{k+1}}$.
  - The distance of the new plates: $y_{k}=\frac{R_{Sin}}{\sin\theta_{k+1}}+\frac{e}{2\tan\theta_{k+1}}$.
5. When $x_{k}\geq {\bar{R}}_{Circle}$ the last level of sinusoid is reached and one poses *n* = *k*. Then in order to be consistent with the direction of the blood flow, the level 1 is defined as the furthest level from the central vein, and the level *n* as the closest one.
6. As a result, the length of each sinusoids level is given by: *L*_*k*_ = min(*y*_*n*−*k*+1_, *R*_*Circle*_) − *x*_*n* − *k*_.

**Table 1:** Lobule parameters.

Parameter	Description	Value
*R*_*Lobule*_	Lobule Radius	790.57μm [14]
*R*_*sin*_	Minimal Sinusoidal Radius	3.65μm [14]
*e*_*Lobule*_	Lobule Thickness	25.00μm [14]
*R*_*H*_	Hepatocyte Radius	8.49μm [14]
*e*	Hepatocyte Plate Width(= 2*R*_*H*_)	16.97μm

Now that the number and the length of the sinusoid levels are defined, the radius within each level changes which is expected to impact the exchange of chemicals between the blood and the hepatocytes. Therefore it is essential to calculate the sinusoid radius changes for every level defined above. The radius along the sinusoids of level *k*, following the blood flow, is then given by:

$$\begin{array}{c} R_{k}(x)=R_{Sin}+(L_{k}-x)\tan\theta_{n-k+1}\;\;\forall x\in [0:L_{k}] \end{array}$$ (1)

Finally, as one assumes that the blood flow *Q*_*k*_ at a given level *k* is identical for all sinusoids, the blood flow and the average velocity are given by:

$$\{\begin{array}{ll} Q_{k} & =\frac{Q_{Lobule}}{6\cdot 2^{n-k}}\\ v_{k}\left(x\right) & =\frac{Q_{k}}{\pi R_{k}{\left(x\right)}^{2}}\forall x\in \left[0:L_{k}\right] \end{array}$$ (2)

where the flow in a lobule is *Q*_*Lobule*_ = *Q*_*Liver*_/*N*_*Lobule*_ with *N*_*Lobule*_ = *V*_*Liver*_/*V*_*Lobule*_ and *V*_*Liver*_ and *V*_*Lobule*_ are, respectively, the liver and lobule volumes. This assumes the same flow in each lobule. Finally, to simplify the notation, in the remaining text we define:

$$\begin{array}{l} \forall x\in \left[0:L_{n}\right]\\ \{\begin{array}{ll} R\left(x\right) & =R_{1}\left(x\right)1_{\left[L_{0}:L_{1}\right]}+\sum_{k=2}^{n}R_{k}\left(x-L_{k-1}\right)I_{]L_{k-1}:L_{k}]}\left(x\right)\\ Q\left(x\right) & =Q_{1}\left(x\right)1_{\left[L_{0}:L_{1}\right]}+\sum_{k=2}^{n}Q_{k}\left(x-L_{k-1}\right)I_{]L_{k-1}:L_{k}]}\left(x\right)\\ v\left(x\right) & =v_{1}\left(x\right)1_{\left[L_{0}:L_{1}\right]}+\sum_{k=2}^{n}v_{k}\left(x-L_{k-1}\right)I_{]L_{k-1}:L_{k}]}\left(x\right) \end{array} \end{array}$$ (3)

where $\forall k\in 〚1:n〛L_{k}=L_{k-1}+L_{k}$, $L_{0}=0$ and *I*_*E*_ is the indicator function of *E*.

### Conservation and kinetic equations for the liver model

#### Conservation equation in the blood

Now that the lobule geometry has been defined, the equations which describe the transport and metabolism of drugs in the liver can be expressed. Before being metabolized in the hepatocytes, the drugs flow with the blood through the lobules and are passively or actively transported into the hepatocytes. Considering *n*_*C*_ drugs and assuming no irreversible reaction within the blood, the conservation equation can be used to describe the concentrations such as:

$$\begin{array}{c} \begin{array}{c} \frac{\partial C_{b}}{\partial t}+v(x)\frac{\partial C_{b}}{\partial x}=-\alpha_{B\to H}(x)[(P+\rho_{in})\cdot f_{u}^{b}\cdot C_{b}-(P+\rho_{out})\cdot f_{u}^{h}\cdot C_{h}] \end{array} \end{array}$$ (4)

where ***C***_***b***_, ***C***_***h***_, ***P***, ***ρ***_***in***_, ***ρ***_***out***_, $f_{u}^{b}$, $f_{u}^{h}$ and *v*(*x*) are, respectively, the concentrations of the drugs in the liver blood and hepatocytes, the permeability and the uptake/efflux rates through the hepatocyte membrane, the fraction unbound in the blood and hepatocytes and the blood velocity; and where *α*_*B* → *H*_(*x*) is the ratio of the elementary blood-hepatocyte surface exchange *δS*_*Exchange*_(*x*) to the elementary blood volume *δV*_*Blood*_(*x*) (see S1 Appendix), given by:

$$\begin{array}{c} \alpha_{B\to H}(x)=\frac{2R(x)+\frac{e_{L}-2R_{H}}{\cos\theta (x)}}{R(x)(e_{L}-2R_{H})} \end{array}$$ (5)

As the blood flow enters the sinusoid from the hepatic arteries at *x* = 0, the initial and boundary conditions are given by:

$$\{\begin{array}{l} C_{b}(x>0,t=0)=0\\ C_{b}\left(x=0,t\right)=C_{0}\left(t\right)\\ C_{h}\left(x,t=0\right)=0 \end{array}$$ (6)

where *C*_0_(*t*) will be defined once the PBPK model will be decribed.

#### Drug kinetic equation in the hepatocytes

**Fig 3:**
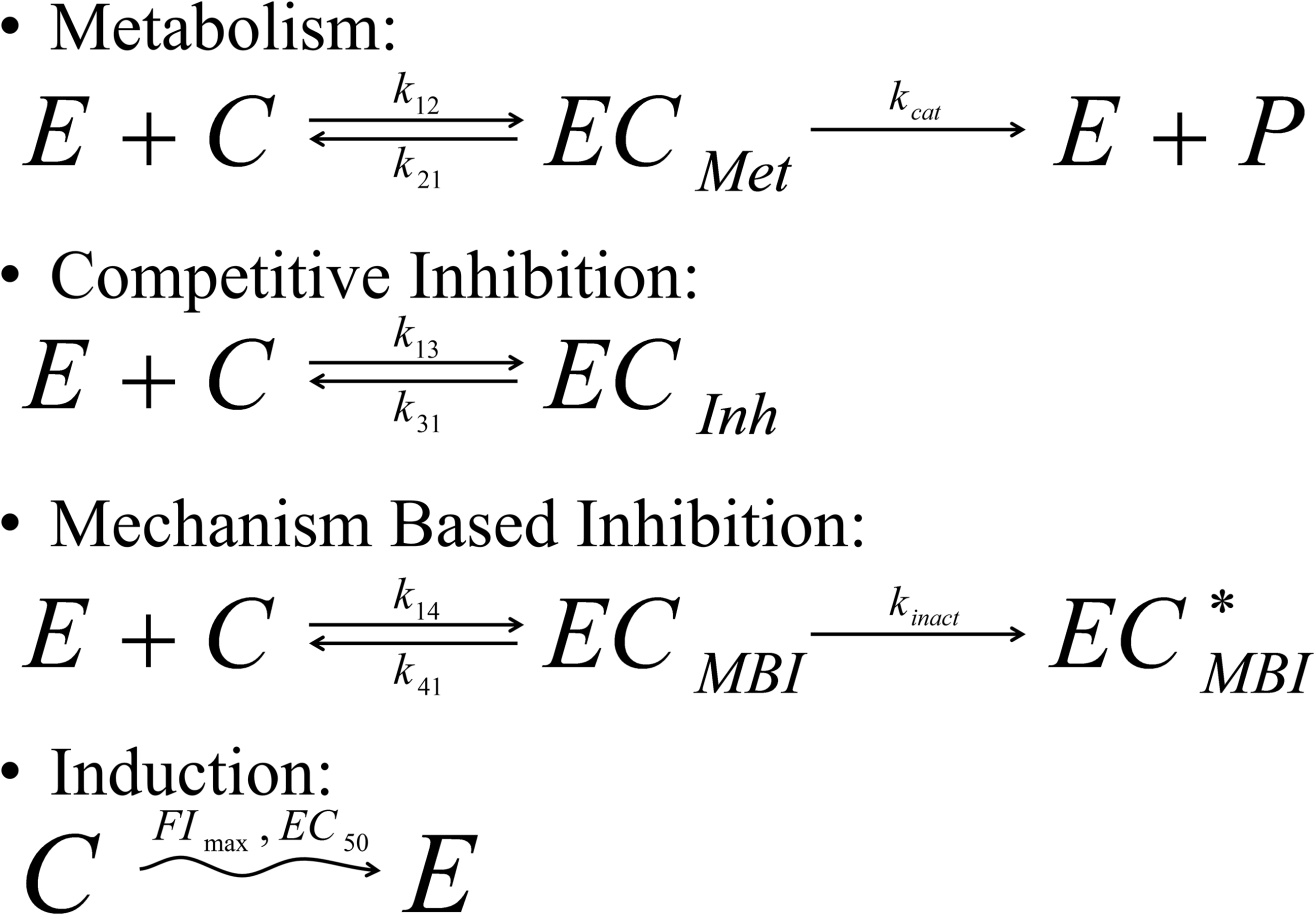
Enzymatic reactions taken into account in the liver model. Reversible inhibition: A drug binds to an enzyme which may result in its metabolism (but not necessarily) resulting in the temporary blockade or inhibition of the enzyme. Here only competitive inhibition will be studied, which assumes that each enzyme can interact with one drug at a time. Mechanism Based Inhibition (MBI): A drug inactivates an enzyme through direct interaction resulting in an inhibited metabolism of any drug metabolized by these enzymes. Induction: A drug induces the expression of one or more enzymes resulting in an induced metabolism of any drug metabolized by these enzymes. Note that the notations in this figure regarding the kinetic rate constants are used in the text.

Once the drugs enter inside the hepatocytes by passive and/or active transport, a cascade of reactions may occur involving metabolism of the drugs by one or more enzymes and includes cross reaction(s) between metabolite(s) and drug(s). The presented model focuses specifically on the reactions schematically represented in Fig 3 (*i*.*e*. Competitive Inhibition, MBI and Induction).

Furthermore, it will be assumed that no exchange of materials between hepatocytes happens and that the equilibrium between the drugs and enzyme complex is quickly reached (see S2 Appendix for the mathematical simplification). Therefore, by using the law of conservation of mass, one can describe the equation governing the concentration of drugs within the hepatocytes by:

$$\begin{array}{c} \begin{array}{cc} \frac{dC_{h}}{dt}= & \alpha_{H\to B}(x)[(P+\rho_{in})\cdot f_{u}^{b}\cdot C_{b}-(P+\rho_{out})\cdot f_{u}^{h}\cdot C_{h}]\\ & -(k_{cat}\cdot EC_{Met})1_{n_{E}}\\ & -(k_{inact}\cdot EC_{MBI})1_{n_{E}}\\ & -\frac{V_{max,2}}{K_{m,2}+f_{u}^{h}\cdot C_{h}}\cdot f_{u}^{h}\cdot C_{h} \end{array} \end{array}$$ (7)

where $EC_{Met}={\left(EC_{Met,i,j}\right)}_{\begin{array}{c} 1\leq i\leq n_{C}\\ 1\leq j\leq n_{E} \end{array}}$ represents the concentration of complex involved in the metabolism of drugs, $EC_{MBI}={\left(EC_{MBI,i,j}\right)}_{\begin{array}{c} 1\leq i\leq n_{C}\\ 1\leq j\leq n_{E} \end{array}}$ the concentration of complex inactivating the enzymes and; ***V*_*max*, 2_** and ***K*_*m*, 2_** the constants associated with unspecified metabolic pathway(s), modeled by a Michaelis-Menten equation. Finally *α*_*H* → *B*_(*x*) is the ratio of the elementary blood-hepatocyte surface exchange *δS*_*Exchange*_(*x*) to the elementary hepatocyte volume *δV*_*Hep*_(*x*) (see S1 Appendix), given by:

$$\begin{array}{c} \alpha_{H\to B}(x)=\frac{2R(x)+\frac{e_{L}-2R_{H}}{\cos\theta }}{R_{H}(2R_{k}(x)+\frac{e_{L}}{\cos\theta })} \end{array}$$ (8)

Eq (7) can be rewritten considering the rapidly attained equilibrium assumption. In this context the enzyme-drug complex concentrations can be expressed as a function of the free enzyme levels and drug concentrations as follow:

$$\begin{array}{c} \begin{array}{c} EC_{Met}=\frac{(f_{u}^{h}\cdot C_{h})E^{tr}}{K_{m,1}}\\ EC_{MBI}=\frac{(f_{u}^{h}\cdot C_{h})E^{tr}}{K_{I}} \end{array} \end{array}$$ (9)

where ***E*** = (*E*_1_, …, *E*_*n*_*E*__)^*tr*^ represents the free enzyme levels and where the constants $K_{m,1}=\frac{k_{21}+k_{cat}}{k_{12}}$ and $K_{I}=\frac{k_{41}+k_{inact}}{k_{14}}$ are developed in S2 Appendix. Therefore the equation becomes:

$$\begin{array}{c} \begin{array}{cc} \frac{dC_{h}}{dt}= & \alpha_{HB}(x)[(P+\rho_{in})\cdot f_{u}^{b}\cdot C_{b}-(P+\rho_{out})\cdot f_{u}^{h}\cdot C_{h}]\\ & -[(k_{cat}/K_{m,1}+k_{inact}/K_{I})E]\cdot f_{u}^{h}\cdot C_{h}-\frac{V_{max,2}}{K_{m,2}+f_{u}^{h}\cdot C_{h}}\cdot f_{u}^{h}\cdot C_{h} \end{array} \end{array}$$ (10)

#### Enzyme kinetic equation in the hepatocytes

The remaining set of equations needs to describe the enzyme kinetics. In general the level of enzymes are assumed to be constant, but when MBI and/or induction occur, changes in enzyme levels are not immediate and time needs to be taken into consideration. Therefore modelling the enzyme kinetics is essential, using classical kinetic equations and assuming that the enzyme induction is additive, the following can be written:

$$\{\begin{array}{l} \begin{array}{ll} \frac{dE_{Tot}}{dt}\approx \frac{dE}{dt}= & k_{deg}\cdot \left[E_{0}+{\left(\frac{E_{max}-1_{n_{C}}E_{0}^{tr}}{EC_{50}+\left(f_{u}^{h}\cdot C_{h}\right)1_{n_{E}}^{tr}}\right)}^{tr}\left(f_{u}^{h}\cdot C_{h}\right)-E_{Tot}\right]\\ & -{\left(k_{inact}\cdot EC_{MBI}\right)}^{tr}1_{n_{C}} \end{array}\\ \frac{dEC_{Met}}{dt}=\frac{dEC_{Inh}}{dt}=\frac{dEC_{MBI}}{dt}\approx 0\\ E_{Tot}=E+{\left[EC_{Met}+EC_{Inh}+EC_{MBI}\right]}^{tr}1_{n_{C}} \end{array}$$ (11)

where $EC_{Inh}={\left(EC_{Inh,i,j}\right)}_{\begin{array}{c} 1\leq i\leq n_{C}\\ 1\leq j\leq n_{E} \end{array}}$ is the concentration(s) of complex that does not metabolize the drugs and is also given by $EC_{Inh}=\frac{(f_{u}^{h}\cdot C_{h})E^{tr}}{K_{i}}$ where $K_{i}=\frac{k_{31}}{k_{13}}$ (see S2 Appendix). The equation above can be further simplified by using Eq (9) and by normalizing the enzyme levels by its initial and basal level *E*_0_ and by noting ${\bar{E}}_{Tot}=E_{Tot}/E_{0}$ and $FI_{max}=E_{max}/\left(1_{n_{C}}E_{0}^{tr}\right)$:

$$\{\begin{array}{l} \frac{d{\bar{E}}_{Tot}}{dt}=k_{deg}\cdot [1+{\left(\frac{\left(FI_{max}-1\right)}{EC_{50}+\left(f_{u}^{h}\cdot C_{h}\right)1_{n_{E}}^{tr}}\right)}^{tr}\left(f_{u}^{h}\cdot C_{h}\right)\\ \;\;\;\;-{\bar{E}}_{Tot}\cdot \left(1+\frac{\frac{1}{k_{deg}}\cdot {\left(\frac{k_{inact}}{K_{I}}\right)}^{tr}\left(f_{u}^{h}\cdot C_{h}\right)}{1+{\left(\frac{1}{K_{m,1}}+\frac{1}{K_{i}}+\frac{1}{K_{I}}\right)}^{tr}\left(f_{u}^{h}\cdot C_{h}\right)}\right)]\\ \bar{E}=\frac{{\bar{E}}_{Tot}}{1+{\left(\frac{1}{K_{m,1}}+\frac{1}{K_{i}}+\frac{1}{K_{I}}\right)}^{tr}\left(f_{u}^{h}\cdot C_{h}\right)} \end{array}$$ (12)

Note that if a drug is not metabolized or does not bind or inactivate a specific enzyme, the related constant is set to infinity, which corresponds to an infinite potency. Furthermore, it is important to note that if two drugs are metabolized by the same enzyme site they automatically inhibit each other and as a result ***K*_*i*_** can be taken as infinity, except if it is suspected that two binding sites are active for a given drug (*e*.*g*. one will metabolize the drug whereas the other will just bind to it). However it is difficult to make this distinction experimentally.

### PBPK model

**Fig 4:**
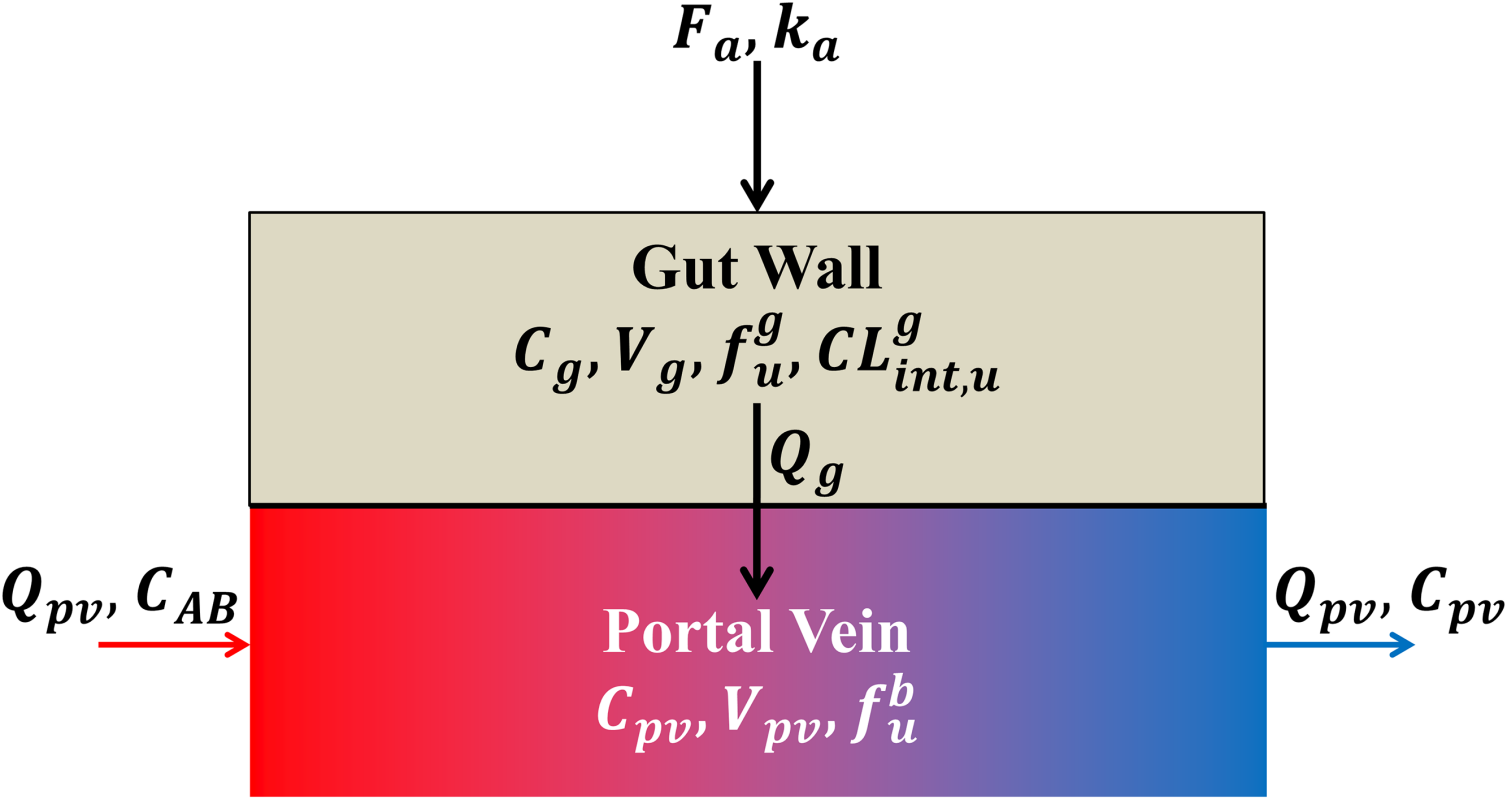
The gut-compartmental model. The gut compartment is composed of two sub-compartments; the gut wall and the portal vein sub-compartments. After an oral administration of a given drug, a fraction ***F*_*a*_** is absorbed from the intestine to the gut wall with an absorption rate constant ***k*_*a*_**. Once the drug is in the gut wall, it may be metabolized and will cross the cell membrane (passively or actively) at a flow *Q*_*g*_, depending on drug permeability and villous blood flow (see S3 Appendix), to join the blood circulatory system. Once the drug is in the blood, it goes to the liver through the portal vein.

Having the liver model defined and the related enzymatic reactions, they need to be incorporated into a PBPK model to be able to simulate the PK of the different drugs and predict their interactions. As seen above, the PBPK model is constituted of 7 compartments: Arterial Blood, Venous Blood, Liver, Gut, Kidneys, Lungs and the Rest of the Body (RB) (Fig 2). All compartments, except the liver and gut, are modeled below as classical compartments associated with their own physiological volume and partition coefficient for drugs [15]. Furthermore, as the drug(s) is(are) administered orally at *t* = 0 the initial concentration of all compartments is taken equal to zero. Finally, each of the compartments is defined as:

- Arterial Blood Compartment:
  $$\begin{array}{c} V_{AB}\frac{dC_{AB}}{dt}=Q_{T}(\frac{C_{Lungs}\cdot R_{BP}}{K_{p,Lungs}}-C_{AB}) \end{array}$$ (13)
  where ***C*_*AB*_** and *V*_*AB*_ are the concentration of drugs and volume of the arterial blood, *Q*_*T*_ the total blood flow and ***C*_*Lungs*_** and ***K*_*p*, *Lungs*_** the concentration and partition coefficient of the lungs; and ***R*_*BP*_** the blood-to-plasma ratio.
- Venous Blood Compartment:
  $$\begin{array}{c} V_{VB}\frac{dC_{VB}}{dt}=Q_{Liver}C_{Liver}+Q_{K}\frac{C_{K}\cdot R_{BP}}{K_{p,K}}+Q_{RB}\frac{C_{RB}\cdot R_{BP}}{K_{p,RB}}-Q_{T}C_{VB} \end{array}$$ (14)
  where ***C*_*VB*_** and ***V*_*VB*_** are the concentration of drugs and volume of the venous blood compartment, ***C*_*Liver*_** and *Q*_*Liver*_ are the concentration of drugs and blood flow for the liver and where, ***C*_*K*_**, ***C*_*RB*_**, *Q*_*K*_, *Q*_*RB*_, ***K*_*p*, *K*_** and ***K*_*p*, *RB*_**, ***C*_*Liver*_** are the concentrations of drugs, the blood flows and partition coefficients of the kidney and the compartment corresponding to the rest of the body (RB-compartment), respectively. To be more specific ***C*_*Liver*_** is the concentration at the exit of the lobule.
- Kidney Compartment:
  $$\begin{array}{c} V_{K}\frac{dC_{K}}{dt}=Q_{K}(C_{AB}-\frac{C_{K}\cdot R_{BP}}{K_{p,K}})-CL_{int,R}\cdot C_{K} \end{array}$$ (15)
  where ***CL*_*int*, *R*_** is the intrinsic renal clearance.
- Lung Compartment:
  $$\begin{array}{c} V_{Lungs}\frac{dC_{Lungs}}{dt}=Q_{Lungs}(C_{VB}-\frac{C_{Lungs}\cdot R_{BP}}{K_{p,Lungs}}) \end{array}$$ (16)
  where *V*_*Lungs*_ and *Q*_*Lungs*_ are the volume of the lungs and blood flow in the lungs, respectively.
- RB-Compartment:
  $$\begin{array}{c} V_{RB}\frac{dC_{RB}}{dt}=Q_{RB}(C_{AB}-\frac{C_{RB}\cdot R_{BP}}{K_{p,RB}}) \end{array}$$ (17)
- Gut Compartment:
  The gut compartment is composed of two sub-compartments [16]; the gut wall and the portal vein sub-compartments (Fig 4). The model to describe the gut wall sub-compartment is similar to the liver model with a few differences including a homogeneous compartment with a first order absorption, differences in enzyme levels and convection to the portal vein. Therefore the concentration of drugs and enzymes within the gut wall are described by:
  $$\{\begin{array}{l} \begin{array}{ll} \frac{dC_{g}}{dt}= & \sum_{i=1}^{n_{Dose}}\frac{F_{a}\cdot D_{i}\cdot k_{a}}{V_{g}}\cdot \text{exp}\left(-k_{a}\left(t-T_{i}\right)\right)H\left(t-T_{i}\right)\\ & -\left[\left(k_{cat}^{g}/K_{m,1}^{g}+k_{inact}^{g}/K_{I}^{g}\right)E_{g}\right]\cdot f_{u}^{g}\cdot C_{g}\\ & -\frac{V_{max,2}^{g}}{K_{m,2}^{g}+f_{u}^{g}\cdot C_{h}}\cdot f_{u}^{g}\cdot C_{g}-\frac{Q_{g}}{V_{g}}f_{u}^{g}\cdot C_{g} \end{array}\\ \begin{array}{ll} \frac{d{\bar{E}}_{Tot,g}}{dt}=k_{deg}^{g} & \cdot [1+{\left(\frac{\left(FI_{max}^{g}-1\right)}{EC_{50}^{g}+\left(f_{u}^{g}\cdot C_{g}\right)1_{n_{E}}^{tr}}\right)}^{tr}\left(f_{u}^{g}\cdot C_{g}\right)\\ & -{\bar{E}}_{Tot,g}\cdot \left(1+\frac{\frac{1}{k_{deg}^{g}}\cdot {\left(\frac{k_{inact}^{g}}{K_{I}^{g}}\right)}^{tr}\left(f_{u}^{g}\cdot C_{g}\right)}{1+{\left(\frac{1}{K_{m,1}^{g}}+\frac{1}{K_{i}^{g}}+\frac{1}{K_{I}^{g}}\right)}^{tr}\left(f_{u}^{g}\cdot C_{g}\right)}\right)] \end{array}\\ {\bar{E}}_{g}=\frac{{\bar{E}}_{Tot,g}}{1+{\left(\frac{1}{K_{m,1}^{g}}+\frac{1}{K_{i}^{g}}+\frac{1}{K_{I}^{g}}\right)}^{tr}\left(f_{u}^{g}\cdot C_{g}\right)} \end{array}$$ (18)
  where ***C*_*g*_** is the concentration of drugs in the gut wall, ***F*_*a*_** the fraction absorbed of the drugs, ***D*_*i*_** the dose at time *T*_*i*_, ***k*_*a*_** the absorption rate constant of the drugs, *n*_*Dose*_ the total number of doses given, $f_{u}^{g}$ the fraction of unbound drugs in the gut wall, *V*_*g*_ the volume of the gut wall, *H* the Heaviside function and ${\bar{E}}_{g}$ and ${\bar{E}}_{Tot,g}$ are the free and total normalized enzyme levels to the initial and basal enzyme level in the gut wall; *E*_0,*g*_. *Q*_*g*_ is a hybrid parameter introduced by Yang [17], which takes into account the membrane permeability of the drugs and blood flow from the enterocytes to the portal vein (see S3 Appendix for more details). All other parameters, except $V_{max,2}^{g}$ and ***E*_0,*g*_**, are taken equal to the corresponding liver values.
  The concentration within the portal vein sub-compartment is given by:
  $$\begin{array}{c} \frac{dC_{pv}}{dt}=\frac{Q_{pv}}{V_{pv}}(C_{AB}-C_{pv})+\frac{Q_{g}}{V_{pv}}\cdot f_{u}^{g}\cdot C_{g} \end{array}$$ (19)
  where ***C*_*pv*_** is the concentration in the portal vein, *Q*_*pv*_ the blood flow of the portal vein and *V*_*pv*_ the volume of the portal vein.
- Liver Compartment:
  Finally the liver compartment is described by the liver model previously described with the boundary condition $C_{0}\left(t\right)=\frac{Q_{ha}C_{AB}\left(t\right)+Q_{pv}C_{pv}\left(t\right)}{Q_{ha}+Q_{pv}}$ at *x* = 0 (Eq (6)). All volumes and blood flows used for the simulation are taken as the average value of a 70 kg man and are summarized in S1 Table

### Numerical resolution

To resolve the herein PBPK, a program was written in Matlab^®^ R2015b [18], using an object-oriented programming (OOP) approach. First, each compartment was identified as a generic object which generates a function ***f*_*i*_** such as $\frac{dY_{i}}{dt}=f(t,Y_{i})$ where ***f*_*i*_** and ***Y*_*i*_** are both column vectors and represent the dynamics of the system and variables of interest (*e*.*g*. blood concentration and enzyme level) of the compartment *i*, respectively. Then, the compartments are combined in a larger object that connects them with their respective blood flows; used to identify the source term for each compartment, and generate a generic function ***f***(*t*, ***Y***) such as $\frac{dY}{dt}=f(t,Y)$ where $f^{tr}=(f_{1}^{tr},f{}_{2}^{tr},\ldots ,f{}_{n}^{tr})$ and $Y^{tr}=(Y{}_{1}^{tr},Y{}_{2}^{tr},\ldots ,Y{}_{n}^{tr})$. Finally, ***Y*** is resolved by using the solver ode15s, which was the preferred solver as it can solve stiff problems and adapt the time step for optimum resolution. A more detailed description of the main architecture of the program can be found in the appendix S6 Appendix and the code with an example in appendix S1 Code.

### Parameters

#### Clinical studies

Ten clinical studies, summarized in Table 2, were selected to assess the predictions from the PBPK model with the observations. For each clinical study, midazolam was used to probe the impact of the perpetrator drug on the CYP3A4 enzyme.

**Table 2:** Summary of 10 *in vivo* clinical studies used in comparison to the simulations.

Perpetrator	Dosage Regimen (p.o)			Victim	Dosage Regimen (p.o)		Observation		Ref.
	Dose	Numbers	Interval		Dose	Intake Time	Ratio		
	(mg)				(mg)	(h)	*AUC* ^a^	*C*_*max*_ ^b^	
Azithromycin	500	3 doses	*q*.*d*.^c^	Midazolam	15	49.5	1.27	1.29	[19]
Cimetidine	400	3 doses	Irregular^d^	Midazolam	15	25	1.35	1.26	[20]
Clarithromycin	500	13 doses	*b*.*i*.*d*.^e^	Midazolam	8	144	8.39	3.80	[21]
Diltiazem	60	5 doses	*t*.*i*.*d*.^f^	Midazolam	15	25	3.75	2.05	[22]
Ethinyl Estradiol	0.03	10 doses	*q*.*d*.	Midazolam	7.5	217	1.20	1.16	[23]
Fluconazole	400	1 doses	*q*.*d*.	Midazolam	7.5	2	3.50	2.50	[24]
Fluoxetine	60 & 20	5 & 7 doses	*q*.*d*.	Midazolam	10	265	0.84	1.11	[25]
Ketoconazole	400	4 doses	*q*.*d*.	Midazolam	7.5	73	15.90	4.09	[26]
Pleconaril	400	15 doses	*t*.*i*.*d*.	Midazolam	5	112	0.65	0.76	[27]
Rifampin	600	10 doses	*q*.*d*.	Midazolam	5.5	Multiple^g^	0.12	0.17	[28]

^*a*^AUC: Area Under the Curve $AUC=\int_{0}^{+\infty }C\left(t\right)dt$

^*b*^Maximum Concentration: $C_{max}=\max_{t\geq 0}C\left(t\right)$

^*c*^*q*.*d*.: *quaque die* (once a day)

^*d*^Intakes at 0, 12 and 24.5 h.

^*e*^*b*.*i*.*d*.: *bis in die* (twice a day)

^*f*^*t*.*i*.*d*.: *ter in die* (three times a day)

^*g*^Intakes at 118 and 190 h.

#### CYP3A4 enzyme

The CYP3A4 enzyme was the enzyme of interest, as it is the main enzyme to metabolize midazolam. The amount of the CYP3A4 enzyme in the liver and intestine was taken as equal to 9.228 μmol [8] and 0.070 μmol [8], respectively. To estimate the concentrations *E*_0_ and $E_{0}^{g}$ for CYP3A4, the respective amounts were divided by the total volume of hepatocytes *V*_*h*_, estimated from the liver model, and the gut wall volume given in S1 Table. The degradation rate constants *k*_*deg*_ and $k_{deg}^{g}$ for CYP3A4 were taken equal to 0.0192 h^−1^ [29] and 0.0288 h^−1^ [29] for the liver and intestine, respectively.

#### Hepatic clearance

In the PBPK model, three parameters, *k*_*cat*_, *V*_*max*,2_ and $V_{max,2}^{g}$, were introduced. The hepatic blood clearance *CL*_*H*_ can easily be obtained from clinical studies, which represents the clearance due to drug metabolism in the liver with respect to the blood compartment. Therefore, *CL*_*H*_ was corrected to estimate the three parameters. This is done first by estimating the apparent intrinsic clearance *CL*_*int*_ assuming a parallel tube model [9], due to the similarity with the herein liver model. Then to correct the impact due to exchange mechanisms between blood and hepatocytes, the metabolic intrinsic clearance $CL_{int}^{*}$ [30] was calculated. The equations for *CL*_*int*_ and $CL_{int}^{*}$ are:

$$\left\{ \begin{array}{l} CL_{int}=-\frac{Q_{Liver}}{f_{u}^{b}}\text{ln}\left(1-\frac{CL_{H}}{Q_{Liver}}\right)\\ CL_{int}^{*}=\frac{S_{ex}\left(P+\rho_{out}\right)CL_{int}}{S_{ex}\left(P+\rho_{in}\right)-CL_{int}} \end{array}\right.$$ (20)

where $f_{u}^{b}$ and *S*_*ex*_ are the blood fraction unbound and the total exchange surface between blood and hepatocytes given by the liver model, respectively. Finally, *k*_*cat*_, *V*_*max*,2_ and $V_{max,2}^{g}$ can be calculated by:

$$\{\begin{array}{ll} k_{cat} & =f_{m,3A4}\cdot \frac{CL_{int}^{*}K_{m,1}}{E_{0}V_{h}}\\ V_{max,2} & =\left(1-f_{m.3A4}\right)\cdot \frac{CL_{int}^{*}K_{m,2}}{V_{h}}\\ V_{max,2}^{g} & =\frac{\left(1-f_{3A4}^{g}\right)A_{CYP}^{g}V_{GW}}{\left(1-f_{3A4}\right)A_{CYP}V_{h}}V_{max,2} \end{array}$$ (21)

where *f*_*m*, 3*A*4_ is the fraction metabolized by the CYP3A4 enzyme, *A*_*CYP*_ and $A_{CYP}^{g}$ are the total amount of CYP in the liver and intestine, respectively, and *f*_3*A*4_ and $f_{3A4}^{g}$ are the fraction amount of CYP3A4 in the liver and intestine, respectively. Hepatic and intrinsic clearances *CL*_*H*_/*CL*_*int*_/$CL_{int}^{*}$ and fraction metabolized *f*_*m*, 3*A*4_ are reported in Table 3, blood fraction unbound $f_{u}^{b}$ in Table 4, and fraction amounts *f*_3*A*4_/$f_{3A4}^{g}$ and CYP amounts *A*_*CYP*_/ $A_{CYP}^{g}$ in Table S2 Table.

**Table 3:** Metabolism parameters of the drugs.

Drug	*CL*_*H*_	*CL*_*m*, *int*_	$CL_{m,int}^{*}$	*K*_*m*_	*f*_*m*, 3*A*4_	*CL*_*R*_
	L/h	L/h	L/h	μM		L/h
Midazolam	34.42 [31]	1095.19	1991.20	2.30 [32]	0.96 [33]	0.09 [5]
Azithromycin	33.60^a^	353.81	392.18	150.00 [29]	1.00^b^	9.29^a^
Cimetidine	13.44 [34]	16.22	16.83	10.00^c^	0.00	17.22^a^
Clarithromycin	26.52 [31]	112.47	131.30	50.00^d^	0.80	6.00 [31]
Diltiazem	50.20^a^^,^^e^	340.21	375.54	30.00^d^	1.00 [35]	2.88^a^
Ethinyl Estradiol	42.52^a^	1643.00	5334.34	18.00^d^	0.60 [36]	0.00
Fluconazole	0.71 [37],^e^	0.80	0.80	10.00^c^	0.00	1.03^a^
Fluoxetine	40.32 [38]	1083.31	1546.61	10.00^c^	0.00	0.00
Ketoconazole	0.69	50.74 [4]	51.46	1.52 [4]	0.00	0.00
Pleconaril	24.29 [39]	1953.50	4248.41	10.00^c^	0.00	0.00
Rifampin	8.66^a^	50.45	51.17	10.00^c^	0.00	1.68^a^

^*a*^Average value from PharmapPendium^®^ database: www.pharmapendium.com

^*b*^DrugBank.

^*c*^Assumed.

^*d*^Assumed to be Equal to *K*_*i*_ when *f*_*m*, 3*A*4_ is not equal to 0.

^*e*^The Hepatic Clearance was estimated by: *CL*_*H*_ = *CL*_*T*_ − *CL*_*R*_

**Table 4:** Fraction unbound and blood-to-plasma ratio of the drugs.

Drug	$f_{u}^{p}$	$f_{u}^{b}$	*R*_*BP*_	$f_{u}^{h}$ ^a^	$f_{u}^{gw}$ ^a^
Midazolam	0.0264	0.0400 [34]	0.66 [31]	0.0202	0.0189
Azithromycin	0.7000 [40]	0.1200 [41]	5.83^b^	0.0031	0.0055
Cimetidine	0.8730	0.9000 [34]	0.97 [42]	0.9880	1.0000
Clarithromycin	0.1800 [31]	0.2813	0.64 [42]	0.0122	0.0984
Diltiazem	0.2028	0.2200 [34]	0.92 [43]	0.0173	0.0251
Ethinyl Estradiol	0.0300^c^	0.0355	0.84^b^	0.0039	0.0023
Fluconazole	0.6893	0.8900 [41]	0.77^b^	0.1051	1.0000
Fluoxetine	0.0500	0.0500 [41]	1.00^d^	0.0057	0.0049
Ketoconazole	0.0095 [43]	0.0136	0.70 [43]	0.0075	0.0048
Pleconaril	0.0100^c^	0.0146	0.69^b^	0.0041	0.0019
Rifampin	0.1100^c^	0.1809	0.61^b^	0.3513	0.2234

^*a*^$f_{u}^{h}=\frac{f_{u}^{p}}{K_{p,Liver}}$ and $f_{u}^{gw}=\frac{f_{u}^{p}}{K_{p,Kidney}}$

^*b*^*R*_*BP*_ = *h* × *K*_*p*, *RBC*_ + 1 − *h* where *h* = *V*_*RBC*_/*V*_*Blood*_ is the hematocrit coefficient.

^*c*^DrugBank database: www.drugbank.ca

^*d*^Assumed.

#### Renal clearance

The renal clearance values *CL*_*R*_ were obtained from the literature (Table 3). As the renal clearance is expressed with respect to the blood compartment, as it is the case for hepatic clearance, an intrinsic renal clearance *CL*_*int*,*R*_ was calculated by assuming a well-stirred model [9]. The intrinsic renal clearance *CL*_*int*,*R*_ can be expressed as:

$$\begin{array}{c} CL_{int,R}=\frac{R_{BP}}{K_{p,K}}\cdot \frac{Q_{K}CL_{R}}{Q_{K}-CL_{R}} \end{array}$$ (22)

where *Q*_*K*_, *R*_*BP*_ and *K*_*p*,*K*_ are the kidney blood flow (S1 Table), the blood-to-plasma ratio (Table 4) and the kidney partition coefficient (Table 5), respectively.

**Table 5:** Tissue-to-plasma partition coefficients of the drugs.

Drug	*K*_*p*, *RBC*_^a^	*K*_*p*, *RB*_^b^	*K*_*p*, *Kidney*_	*K*_*p*, *Lungs*_	*K*_*p*, *Liver*_	*K*_*p*, *Gut*_
Midazolam	0.005^c^	0.84 [44]	1.41 [44]	1.61 [44]	1.31 [44]	1.40 [44]
Azithromycin	12.424^c^	77.34^c^	110.75^c^	23.16^c^	226.15^c^	126.72^c^
Cimetidine	0.657^c^	0.74^c^	0.88^c^	0.89^c^	0.88^c^	0.83^c^
Clarithromycin	0.252^c^	1.51^c^	1.02^c^	0.43^c^	1.24^c^	1.83^c^
Diltiazem	0.816^c^	5.39^c^	6.26^c^	1.49^c^	11.76^c^	8.10^c^
Ethinyl Estradiol	0.632^c^	10.93^c^	6.11^c^	1.38^c^	7.73^c^	12.79^c^
Fluconazole	0.467^c^	0.54^c^	0.65^c^	0.63^c^	0.66^c^	0.68^c^
Fluoxetine	1.000^c^	6.31^c^	8.96^c^	1.87^c^	18.28^c^	10.30^c^
Ketoconazole	0.096^c^	1.75^c^	1.01^c^	0.39^c^	1.20^c^	2.00^c^
Pleconaril	0.258^c^	4.65^c^	2.58^c^	0.69^c^	3.22^c^	5.34^c^
Rifampin	0.074^c^	0.46^c^	0.32^c^	0.29^c^	0.31^c^	0.49^c^

^*a*^RBC: Red Blood Cells.

^*b*^Estimated by averaging the partition coefficients of the remaining tissues: $K_{p,RB}=\sum_{i=1}^{n}V_{i}K_{p,i}/\sum_{i=1}^{n}V_{i}$(see S5 Appendix for equation development).

^*c*^Theoretical values estimated using the equations by Rodgers and Rowland. Two formula were used; one for the moderate to strong bases (pK_a_ > 7) and the group 1 zwitterions (pK_a,1_ > 7) (Rodgers *et al*. 2005) and the second for acids, neutrals, weak bases (pK_a_ < 7) and group 2 zwitterions (pK_a,1_ < 7) (Rodgers *et al*. 2006). The parameters used in these equations are given in S3 and S4 Tables.

#### Remaining parameters

The partition coefficients for each compartment and drug are found in Table 5. Parameters related to reversible inhibition, MBI and induction are reported in Table 6 for each drug, whereas fraction absorbed *F*_*a*_, absorption constant rate *k*_*a*_, the hybrid gut wall flow *Q*_*g*_, and the permeability *P* for each drug are shown in Table 7. Finally, it is assumed that there is no active hepatocyte uptake or efflux for all drugs considered in this present work (*i*.*e*.*ρ*_*in*_ = 0 and *ρ*_*out*_ = 0).

**Table 6:** Interaction parameters of the drugs.

Drug	Inhibition	MBI		Induction		
	*K*_*i*_	*k*_*inact*_	*K*_*I*_	*FI*_*max*_	*EC*_50_	$EC_{50}^{*}$ ^a^
	μM	h^−1^	μM		μM	μM
Azithromycin	150.00 [29]	0.30 [7]	19.00 [7]	1^b^	+∞^b^	+∞
Cimetidine	115.00 [29]	0^b^	+∞^b^	1^b^	+∞^b^	+∞
Clarithromycin	50.00 [29]	3.18 [29]	18.90 [29]	1^b^	+∞^b^	+∞
Diltiazem	30.00 [29]	1.68 [29]	1.15 [29]	1^b^	+∞^b^	+∞
Ethinyl Estradiol	18.00 [7]	2.40 [7]	18.00 [7]	70.00 [7]	20.00 [7]	3.33
Fluconazole	3.40 [29]	0^b^	+∞^b^	1^b^	+∞^b^	+∞
Fluoxetine	8.00 [29]	[29]	0.61 [29]	3.10 [29]	0.54 [29]	0.18
Ketoconazole	0.006 [29]	0^b^	+∞^b^	1^b^	+∞^b^	+∞
Pleconaril	+∞	0^b^	+∞^b^	34.00 [45]	16.40 [45]	3.83
Rifampin	100.00 [29]	0^b^	+∞^b^	34.00 [29]	0.57 [29]	0.54

^*a*^*EC*_50_ was corrected to take into account fraction unbound in incubation, permeability and active transport where: $EC_{50}^{*}=f_{u,inc}\frac{S_{ex}(P+\rho_{in})}{S_{ex}(P+\rho_{out})+CL_{int}^{*}}\times EC_{50}$, *S*_*ex*_ = 10046 dm^2^ is given by the liver model and *f*_*u*, *inc*_ is calculated theoretically by using the formula by Kilford *et al*. 2008.

^*b*^Not known as being a reversible inhibitor, MBI inhibitor or inducer. If not a reversible inhibitor *K*_*i*_ = +∞, if not a MBI inhibitor *k*_*inact*_ = 0 and *K*_*I*_ = +∞ and if not an inducer *FI*_*max*_ = 1 and *EC*_50_ = +∞.

**Table 7:** Fraction absorbed, absorption constant rate, *Q*_*g*_ and permeability of drug chemicals.

Drug	*F*_*a*_	*k*_*a*_	*Q*_*g*_^a^	*P*
		h^−1^	L/h	μm/h
Midazolam	1.00^b^	1.16 [31]	15.44	24228.0 [15]
Azithromycin	0.86 [31]	0.11 [31]	20.51	36000.0^b^
Cimetidine	1.00^b^	1.00^b^	2.57	4468.6 [46]
Clarithromycin	0.55 [47]	1.08 [31]	4.77	7807.8 [48]
Diltiazem	1.00 [8]	1.60 [8]	18.41	36000.0^b^
Ethinyl Estradiol	1.00^b^	1.00^b^	15.13	23635.9 [36]
Fluconazole	0.86 [37]	0.88 [37]	6.23	13646.5 [46]
Fluoxetine	1.00^b^	1.00^b^	22.29	36000.0^b^
Ketoconazole	1.00^b^	1.00^b^	23.34	36000.0^b^
Pleconaril	0.70^c^	1.00^b^	23.31	36000.0^b^
Rifampin	1.00^b^	1.00^b^	19.18	36000.0^b^

^*a*^$Q_{g}=\frac{CL_{perm}\frac{Q_{v}}{f_{u}^{b}}}{CL_{perm}+\frac{Q_{v}}{f_{u}^{b}}}$: see S3 and S4 Appendices for more details.

^*b*^Assumed.

^*c*^DrugBank database: www.drugbank.ca

## Results

### Algorithm construction of liver

The algorithm to construct the lobule geometry generated 5 sinusoidal levels, where the length of each level is represented in Table 8. The volume of one lobule and the number of lobules, estimated from the parameters in Table 1, are respectively 4.06 × 10^7^ μm^3^ and 4.16 × 10^7^. Given the average liver volume of 1.69 L for a man of 70 kg (S1 Table), the construction of the lobules respecting the algorithm presented in Fig 1C gave a total hepatocytes volume *V_h_* = 1392 mL and blood volume *V_b_* = 283 mL. Therefore the liver volume given by the model is 1.67 L, which is slightly less than the input volume. Similarly the blood content can be compared to the literature which varies between 250 mL and 312 mL [49]. Furthermore, the surface exchange between blood and hepatocytes *S*_*ex*_ given by the model was estimated to be 10046 dm^2^, which is expected to influence the calculation of $CL_{int}^{*}$ from *in vivo* data. Finally, assuming a cell volume of *V*_*cell*_ = 4 μL/10^6^ cells [50], the number of hepatocytes per liver is 348 × 10^9^ cells which is equivalent to 193 × 10^6^ cells/g of liver. This compares well to the literature values which range from 65 to 185 × 10^6^ cells/g of liver [51].

**Table 8:** Sinusoids length for each level.

Level	Length
	μm
1	344.8
2	185.3
3	92.5
4	46.0
5	22.5
**Total**	**691.1**

**Fig 5:**
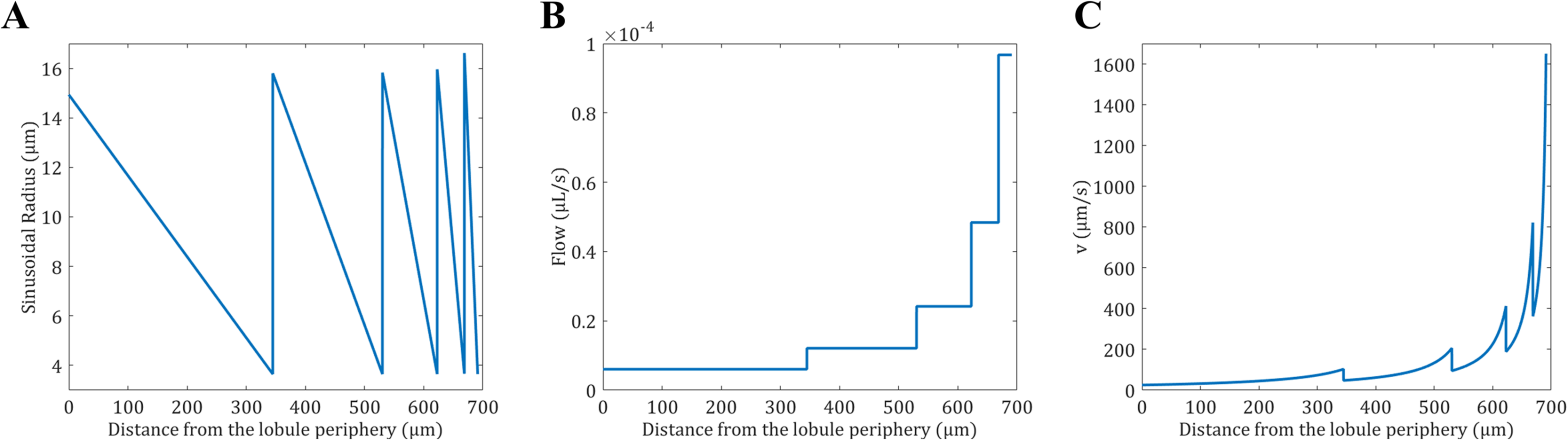
Properties of 5 sinusoid levels from the lobule model. (A) The radius of the sinusoids is expressed as a function of the distance to the periphery of the lobule. For a given level, the radius is decreasing as the sinusoids are converging toward the center of the lobule. Once the sinusoids reach their minimum size they merge together which increases the radius size in a stepwise manner. (B) The flow of the sinusoids is expressed as a function of the distance to the periphery of the lobule. For a given level, the flow is constant, but double when two sinusoids merge. (C) The velocity of the sinusoids is expressed as a function of the distance to the periphery of the lobule. For a given level, the velocity is increasing as the sinusoid radius is decreasing. Once the sinusoids reach their minimum size they merge which decreases the blood velocity suddenly.

The mathematical construction of the lobule gives the radius of the sinusoids for each level as represented in Fig 5A. Given the number of lobules and the blood flow in the liver, the blood flow for each sinusoid level can be estimated by dividing the total blood flow by the total number of sinusoids at a given level as represented in Fig 5B. From the liver blood flow and the radius of the sinusoids, the velocity in the sinusoids (Fig 5C) was calculated as the ratio of the sinusoids flow to the cross section area as follows: *v*(*x*) = *Q*(*x*)/*S*(*s*), where the cross section area of the sinusoids is expressed as *S*(*x*) = *R*(*x*) (*e*_*L*_ − 2*R*_*H*_) (see S1 Appendix).

### Simulations and comparisons

**Fig 6:**
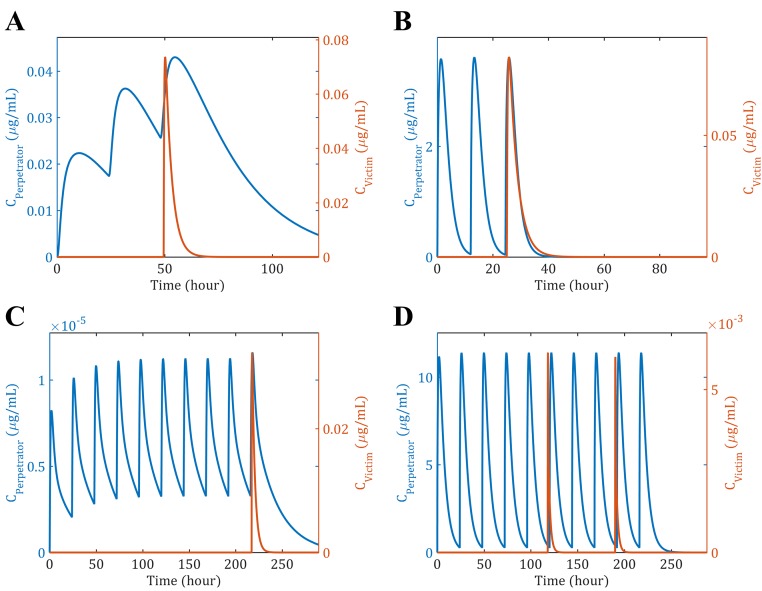
Simulated PK of the perpetrator (blue) and victim (orange) drugs. The simulation were run using the clinical dose regimens from Table 2: (A) Azithromycin (B) Cimetidine (C) Ethinyl Estradiol (D) Rifampin.

**Fig 7:**
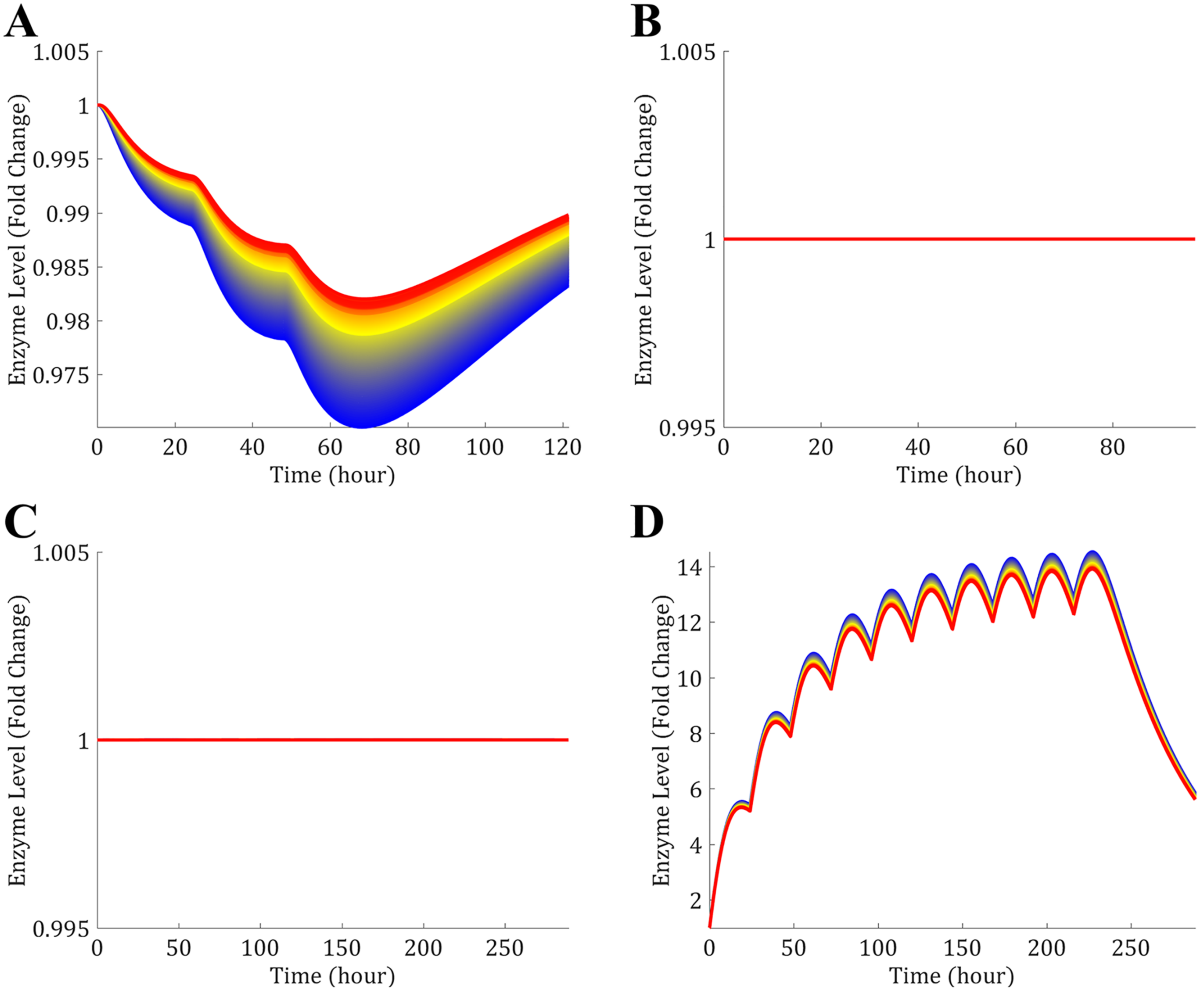
Simulated enzyme levels as a function of time. The total enzyne level (free enzyne + enzyme-substrate complex) is represented as a fold change compared to the initial level. The color gradient indicates the positions within the lobule from blue (Entrance of the lobule) to red (Exit of the lobule): (A) Azithromycin (MBI inducer) (B) Cimetidine (Reversible inhibitor: No effect on enzyme level) (C) Ethinyl Estradiol (MBI inhibitor and inducer: It seems that in this case the effect cancels each other out) (D) Rifampin (Inducer).

**Fig 8:**
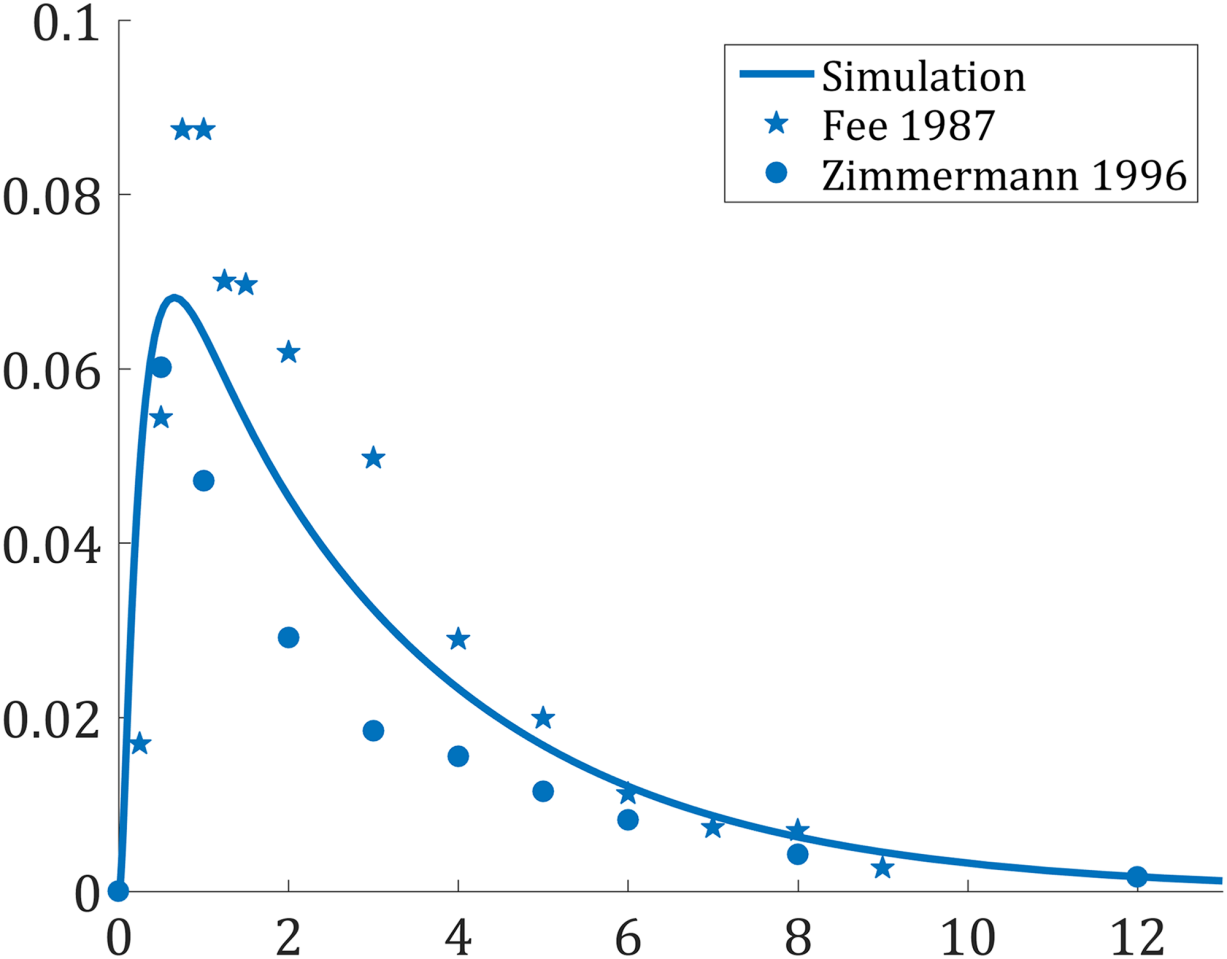
Simulated PK profile for midazolam after an oral dose of 15 mg and comparison to clinical data. (⋆) Fee *et al*. 1987 [20] (•) Zimmermann *et al*. 1996 [19].

**Fig 9:**
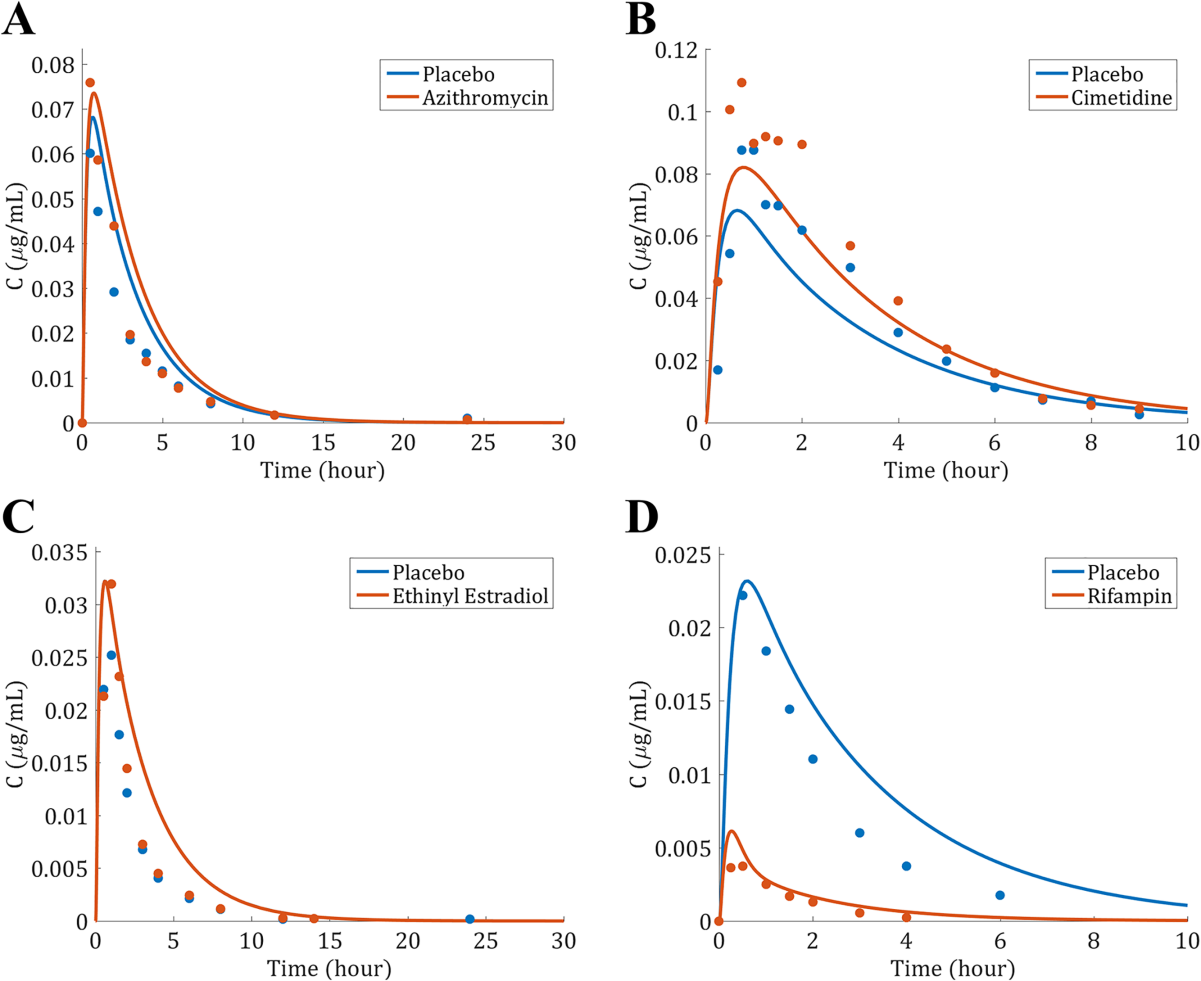
Simulated PK profiles for midazolam with a placebo (blue) or a perpetrator (orange) and comparison to clinical data. The dots represent the clinical observations: (A) Azithromycin [19] (B) Cimetidine [20] (C) Ethinyl Estradiol [23] (D) Rifampin [28].

**Fig 10:**
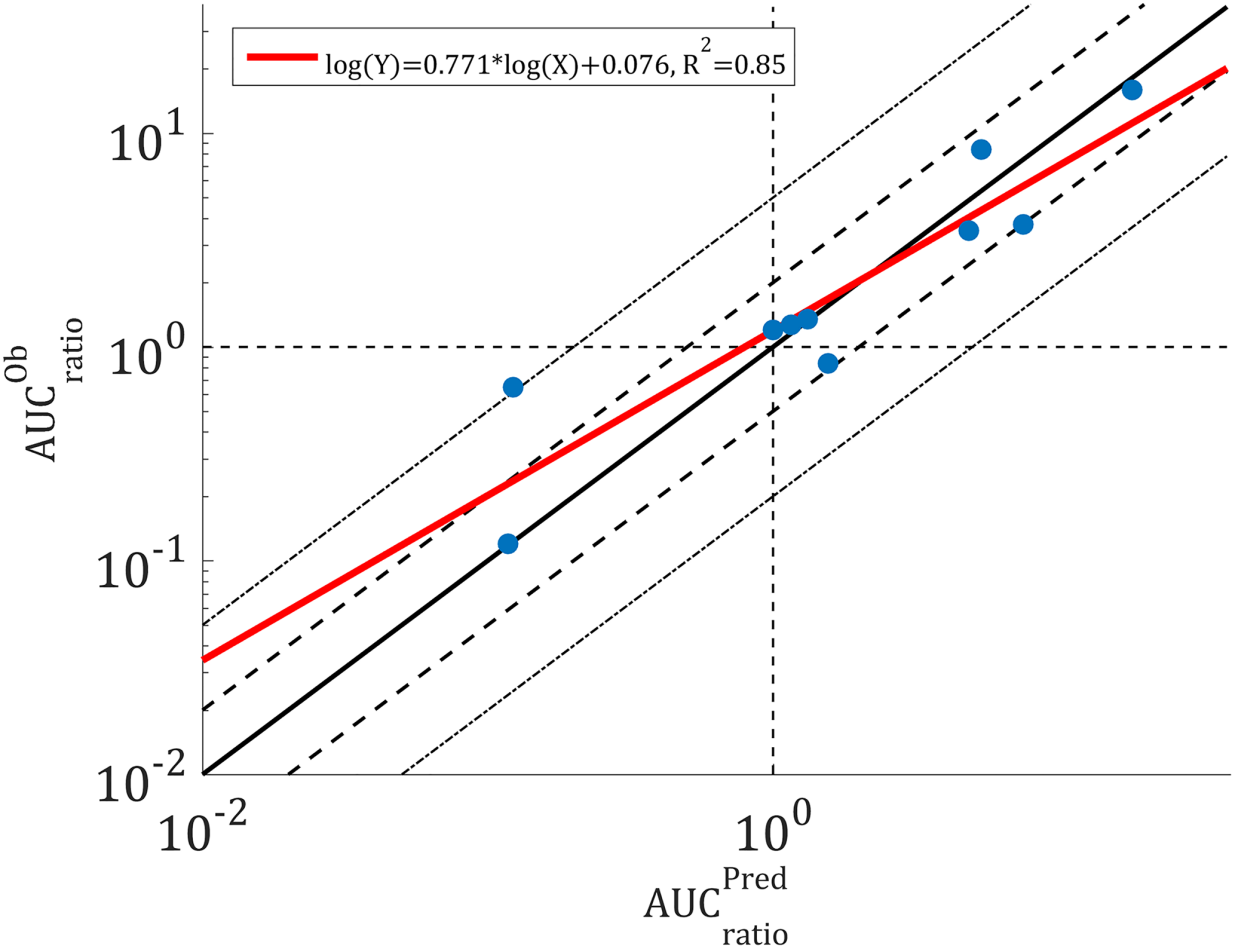
Observed *AUC*_*ratio*_ versus predicted *AUC*_*ratio*_. The solid line represents the line of unity, the dashed lines are the 2-fold errors and the dotted lines the 5-fold errors.

The clinical data presented in Table 2 were simulated and the PK profiles of the victim and perpetrator drugs are represented in Fig 6. In addition to the PK profiles, the model simulates the enzyme level in the liver and gut wall as a result of the different mechanisms involved in drug metabolism. The enzyme levels as a function of time in the liver are given in Fig 7, where the spatial effect is also represented with a color gradient. Furthermore, the PK profile of midazolam alone was simulated and compared to clinical data in Fig 8. The model seems to adequately predict midazolam PK. Finally, the PK profiles of midazolam with a placebo and the perpetrator for each of the clinical studies in Table 2 are represented in Fig 9 and a comparison of the prediction and clinical observation of the *AUC*_*ratio*_ and *C*_*max*,*ratio*_ are summarized in Table 9. Fold error in eight out of ten predictions are within 2-fold which is a common criteria for good prediction [29, 52]. In Fig 10 the observed *AUC*_*ratio*_ are plotted against the predicted *AUC*_*ratio*_. Most of the predictions are relatively well aligned with the line of unity except for the pleconaril scenario where the induction was overpredicted, therefore *AUC*_*ratio*_ underpredicted. An *R*^2^ of 0.85 was calculated which indicates a good correlation between the observations and the model predictions.

**Table 9:** DDI prediction for the 10 clinical studies.

Drug		*AUC*_*ratio*_			*C*_*max*, *ratio*_		
*Perpetrator*	*Victim*	*Observation*	*Prediction*	*F*.*E*.^a^	*Observation*	*Prediction*	*F*.*E*.^a^
Azithromycin	Midazolam	1.27	1.16	1.10	1.29	1.08	1.20
Cimetidine	Midazolam	1.35	1.32	1.02	1.26	1.20	1.04
Clarithromycin	Midazolam	8.39	5.36	1.57	3.80	2.24	1.69
Diltiazem	Midazolam	3.75	7.52	2.01	2.05	2.37	1.16
Ethinyl Estradiol	Midazolam	1.20	1.00	1.20	1.16	1.00	1.16
Fluconazole	Midazolam	3.50	4.85	1.38	2.50	2.15	1.16
Fluoxetine	Midazolam	0.84	1.56	1.85	1.11	1.27	1.14
Ketoconazole	Midazolam	15.90	18.17	1.14	4.09	3.09	1.33
Pleconaril	Midazolam	0.65	0.12	5.30	0.76	0.24	3.17
Rifampin	Midazolam	0.12	0.12	1.02	0.17	0.26	1.56
			**GMFE**^b^	**1.52**		**GMFE**^b^	**1.38**

^*a*^$FoldError=10^{\log|\frac{Ob}{Pred}|}$

^*b*^GMFE: Geomtric Mean Fold Error $GMFE=10^{\sum_{i=1}^{N}\log|\frac{Ob_{i}}{Pred_{i}}|}$

## Discussion

The objective of this work was to develop a mathematical model to predict PK drug-drug interactions in a dynamic manner which may occur in the liver and the intestine. The main focus was on the liver, as the majority of drug metabolism and therefore DDIs occur in this organ. However, to incorporate first pass metabolism the intestine was also included. The main results from this work are; (i) the liver model is capable of describing the geometry of a lobule in a simple manner; (ii) the liver model can be incorporated into a PBPK model to predict the PK profile of a drug; (iii) the PBPK model is, so far, capable of predicting the DDIs when one enzyme is mainly involved in DDIs. The novelty of the model presented in this work is the description of the lobule/liver geometry in the simplest manner possible to account for spatial variation in blood flow, concentrations and enzyme level. Furthermore, a cellular model was included to model drug transport, *i*.*e*. permeability, uptake and efflux, between blood and hepatocytes and drug metabolism within the hepatocytes, without creating a discontinuity with the historical models (*e*.*g*. the well stirred model [9] or parallel tube model [9]). It has the advantage of comparing the geometrical properties of the lobule generated by the model to physiological data such as the liver blood content, number of lobules, surface exchange, sinusoidal radius, velocity, blood flow profile and, the hydrodynamic pressure load. More detailed geometries have been proposed [14, 53, 54], but their implementation into a pharmaceutical context is not optimal as it is demanding in computational resources (computing power and scientific IT support). Therefore it seems that the liver model herein is an appropriate compromise between the complexity of the model and its implementation. The calculated blood content in the liver (excluding arteries and veins) is in the range of literature values which varies between 250 mL and 312 mL [49]. However, the number of cells per gram of liver is relatively higher than the literature values which range from 65 to 185 × 10^6^ cells/g of liver [51]. As it was assumed that the hepatocyte plates are homogeneous, the space of Disse and other cell types (*e*.*g*. Kupffer cells) were neglected which could have lead to an overestimation of the number of hepatocytes. Based on the lobule geometry, the surface estimated for exchanges *S*_*ex*_ might have been underestimated as it is half of the maximum surface of exchange; assuming that the surface of all the cells is in contact with blood, which in turn is expected to influence the calculation of the metabolic intrinsic $CL_{int}^{*}$ obtained from *in vivo* data for low permeable drugs. A comparison of *in vivo* and *in vitro* data across a range of drugs may allow to estimate a more realistic value of *S*_*ex*_. Finally, the pharmacokinetic profile of midazolam was relatively well predicted as well as the impact of the perpetrator drug on its *AUC* and *C*_*max*_. Indeed the *GMFE*_*AUC*_ was estimated to 1.52 which is in the lower range of literature values (1.47–2.5 [7, 29]). A comparison to the static combined model by Fahmi *et al*. [7] and to a well-stirred model similar to the DDI model by Rowland-Yeo *et al*. [8] was also made (results not shown) where the *GMFE*_*AUC*_ were estimated at 2.55 and 1.71, respectively, which suggests that dynamic models are far superior to static models and that geometry might help to improve predictions. However, it is worth noting that the herein results were estimated without taking into account hepatic uptake, which generally improves predictions [55], and without fitting any parameters. All parameters were taken from the literature or calculated using published algorithms. Ideally, each parameter should be estimated experimentally in a specific *in vitro* assays, where it is assumed that they are representative of what is happening *in vivo*. This point needs careful consideration as measuring the drug-metabolizing ability of isolated hepatocytes leads very often to under-predictions of drug clearance. Moreover, studies have shown that an oxygen gradient [56, 57] and blood flow [58] (*i*.*e*. shear stress) affect the expression levels of CYPs. The liver model shows that the blood flow inside the liver is non-linear due to its hierarchical anatomical structure and may explain the notion of zonation, *i*.*e*. CYPs are more highly expressed in certain zones of lobules compared to others. Both effects could be incorporated into the model, where the oxygen concentration and the variation in shear stress, related to changes in velocity in a lobule, can be modeled.

## Conclusion

A liver model including a simple description of the lobule geometry and the uptake/efflux transport between the blood and hepatocytes was presented. The model predicts the pharmacokinetic profiles, enzyme activity and drug-drug interaction for different type of DDIs. Future research will test the model with two or more enzymes involved in metabolism to validate the model further, take into consideration uncompetitive, non-competitive or mixed inhibition and potentially add a component for the biliary excretion which is not negligible for some drugs. Furthermore, the model needs to be compared to models with increasing complexity, *i*.*e*. from static models to dynamic model, to assess how the new features of the herein model improves DDI predictions. Finally, this research focused on the liver as it is the main organ involved in drug metabolism, but the intestine and kidneys may play a significant role in DDIs. Therefore combining the herein liver model to a more sophisticated gut model (*e*.*g*. the advanced compartmental absorption and transit (ACAT) model) and/or a kidney model, where transporters are taking into account, could potentially improve the prediction of DDIs in the future.

## Supporting information

S1 Appendix The geometry of sinusoids. Description of how the the parameters *α*_*B* → *H*_(*x*) and *α*_*H* → *B*_(*x*) were obtained.
(PDF)

S2 Appendix Model simplification. Detailed descriptions on how Eqs (10) and (12) were obtain from Eqs (7) and (11).
(PDF)

S3 Appendix The hybride parameter *Q*_*g*_. The two definitions of the parameter *Q*_*g*_ by Yang *et al*. [17] and Hisaka *et al*. [16] are presented.
(PDF)

S4 Appendix Hisaka equation for *Q*_*g*_. Description of how *Q*_*g*_ is deduced by Hisaka *et al*. [16].
(PDF)

S5 Appendix Average partition coefficient. Description of how the partition coefficient of a PK compartment composed of different tissues is calculated.
(PDF)

S6 Appendix Numerical resolution and OOP. A brief description of Object-Oriented Programming (OOP) and a detail description on the program to solve the PBPK model.
(PDF)

S1 Table Average volume and blood flow for a 70 kg man for different tissues. (PDF)

S2 Table Amounts and degradation rate constants for different CYP enzymes in the liver and the intestine. (PDF)

S3 Table Physico-chemical properties of the drugs. Physico-chemical parameters used to calculate the partition coefficients.
(PDF)

S4 Table Composition of human tissue for different organs. Tissue composition used to calculate the partitions coefficient.
(PDF)

S1 Code Matlab code. The code of all the objects used for the simulations and an example on how to use them to solve a PBPK model.
(RAR)
