# Supplementary material for: A quantitative systems pharmacology approach, incorporating a novel liver model, for predicting pharmacokinetic drug-drug interactions
Source: PLoS One. 2017 Sep 14;12(9):e0183794. doi: 10.1371/journal.pone.0183794 (PMC5598964; doi:10.1371/journal.pone.0183794)
Supplement: S1 Appendix — Description of how the the parameters αB → H(x) and αH → B(x) were obtained. (PDF) [file pone.0183794.s001.pdf]

S1 Appendix: Geometry of Sinusoids

In Eq (1) and (4), two terms $\alpha_{B\to H}(x)$ and $\alpha_{H\to B}(x)$ were introduced. These two terms are simply representative of the sinusoidal geometry and are defined as the ratio of the elementary blood-hepatocyte exchange surface $\delta S_{Exchange}$ to the elementary blood volume δV_{Blood} and the ratio of $\delta S_{Exchange}$ to the elementary hepatocyte volume δV_{Hep}. Therefore before expressing $\alpha_{B\to H}(x)$ and $\alpha_{H\to B}(x)$, one will need to express $\delta S_{Exchange}$, δV_{Blood} and δV_{Hep}. Considering the algorithm to construct a lobule described in Fig 1C and the representation in Fig S1.1 one can show:

$$\begin{cases} \delta S_{Exchange}(x,dx) & = 2\dfrac{2R(x)+2R(x+dx)}{2}dx + 2\dfrac{dx}{\cos\theta(x)}(e_L - 2R_H) \\ \delta V_{Blood}(x,dx) & = (e_L - 2R_H)\dfrac{2R(x)+2R(x+dx)}{2}dx \\ \delta V_{Hep}(x,dx) & = 2R_H\dfrac{2R(x)+2R(x+dx)}{2}dx + 2R_H\dfrac{e_L}{\cos\theta(x)}dx \end{cases} \tag{S1.1}$$

where $R(x+dx)$ can be expressed as $R(x+dx) = R(x) - \tan\theta(x)\,dx$ and therefore the formula can be simplified as:

$$\begin{cases} \delta S_{Exchange}(x,dx) & = 2\left(2R(x) - \tan\theta(x)\,dx + \dfrac{e_L - 2R_H}{\cos\theta(x)}\right)dx \\ \delta V_{Blood}(x,dx) & = (e_L - 2R_H)(2R(x) - \tan\theta(x)\,dx)\,dx \\ \delta V_{Hep}(x,dx) & = 2R_H\left(2R(x) - \tan\theta(x)\,dx + \dfrac{e_L}{\cos\theta(x)}\right)dx \end{cases} \tag{S1.2}$$

Now that the elementary surface and volumes are expressed, $\alpha_{B\to H}(x)$ and $\alpha_{H\to B}(x)$ can finally be defined as:

$$\begin{cases} \alpha_{B\to H}(x) & = \lim\limits_{dx\to 0}\dfrac{\delta S_{Exchange}}{\delta V_{Blood}} = \dfrac{2R(x) + \frac{e_L - 2R_H}{\cos\theta(x)}}{R(x)(e_L - 2R_H)} \\ \alpha_{H\to B}(x) & = \lim\limits_{dx\to 0}\dfrac{\delta S_{Exchange}}{\delta V_{Hep}} = \dfrac{2R(x) + \frac{e_L - 2R_H}{\cos\theta(x)}}{R_H\left(2R_k(x) + \frac{e_L}{\cos\theta(x)}\right)} \end{cases} \tag{S1.3}$$

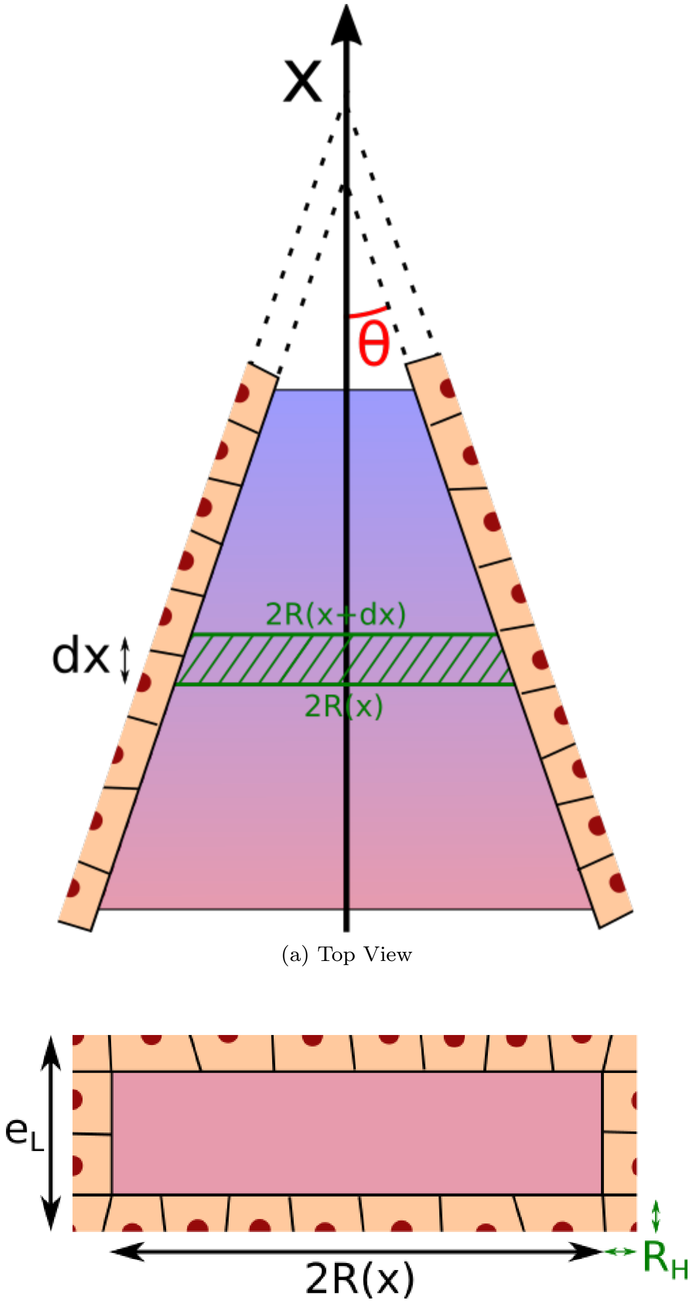

(a) Top View

(b) Cross Section

Figure S1.1: Geometry Representation of a sinusoid
