# Supplementary material for: A quantitative systems pharmacology approach, incorporating a novel liver model, for predicting pharmacokinetic drug-drug interactions
Source: PLoS One. 2017 Sep 14;12(9):e0183794. doi: 10.1371/journal.pone.0183794 (PMC5598964; doi:10.1371/journal.pone.0183794)
Supplement: S2 Appendix — Detailed descriptions on how Eqs (10) and (12) were obtain from Eqs (7) and (11). (PDF) [file pone.0183794.s002.pdf]

S2 Appendix: Model Simplification

In this section, Eq (7) and (9), which describes the kinetics of the drugs and enzymes in the hepatocytes assuming fast equilibrium, respectively, will be fully developped.

S2.1 Drugs

After the drugs have been passively or actively transported into the hepatocytes, they may be metabolized by one or more pathways (see Fig S2.1). This is described by:

$$\begin{aligned}\frac{d\boldsymbol{C_h}}{dt} &= \alpha_{H\to B}(x)\left[(\boldsymbol{P}+\boldsymbol{\rho_{in}}).\boldsymbol{f_u^b}.\boldsymbol{C_b} - (\boldsymbol{P}+\boldsymbol{\rho_{out}}).\boldsymbol{f_u^h}.\boldsymbol{C_h}\right] \\ &- \left[\boldsymbol{k_{12}}.\left(\left(\boldsymbol{f_u^h}.\boldsymbol{C_h}\right)\boldsymbol{E}^{tr}\right) - \boldsymbol{k_{21}}.\boldsymbol{EC_{Met}}\right]\mathbb{1}_{\boldsymbol{n_E}} \\ &- \left[\boldsymbol{k_{13}}.\left(\left(\boldsymbol{f_u^h}.\boldsymbol{C_h}\right)\boldsymbol{E}^{tr}\right) - \boldsymbol{k_{31}}.\boldsymbol{EC_{Inh}}\right]\mathbb{1}_{\boldsymbol{n_E}} \\ &- \left[\boldsymbol{k_{14}}.\left(\left(\boldsymbol{f_u^h}.\boldsymbol{C_h}\right)\boldsymbol{E}^{tr}\right) - \boldsymbol{k_{41}}.\boldsymbol{EC_{MBI}}\right]\mathbb{1}_{\boldsymbol{n_E}} \\ &- \frac{\boldsymbol{V_{max,2}}}{\boldsymbol{K_{m,2}}+\boldsymbol{f_u^h}.\boldsymbol{C_h}}.\boldsymbol{f_u^h}.\boldsymbol{C_h}\end{aligned} \tag{S2.1}$$

where $\boldsymbol{E} = (E_1, \ldots, E_{n_E})^{tr}$ represents the enzymes levels, $\boldsymbol{EC_{Met}} = (EC_{Met,i,j})_{\substack{1\le i\le n_C \\ 1\le j\le n_E}}$ the concentration of complex which can metabolize the drugs, $\boldsymbol{EC_{Inh}} = (EC_{Inh,i,j})_{\substack{1\le i\le n_C \\ 1\le j\le n_E}}$ the concentration of complex that does not metabolize the drugs, $\boldsymbol{EC_{MBI}} = (EC_{MBI,i,j})_{\substack{1\le i\le n_C \\ 1\le j\le n_E}}$ the concentration of complex which can inactivate the enzyme and; $\boldsymbol{V_{max,2}}$ and $\boldsymbol{K_{m,2}}$ the constants associated with unspecified metabolic pathway(s), modeled by a Michaelis-Menten equation. Furthermore, the kinetics constants $\mathbf{k_{12}}$, $\mathbf{k_{21}}$, $\mathbf{k_{cat}}$, $\mathbf{k_{13}}$, $\mathbf{k_{31}}$, $\mathbf{k_{14}}$, $\mathbf{k_{41}}$ and $\mathbf{k_{inact}}$ describe the enzymatic reactions showed in Fig S2.1 and $\alpha_{H\to B}(x)$ has been described in S1 Appendix.

Assuming that the equilibrium between the drugs and enzyme complex is quickly reached (see appendix S2.2), one can show that:

$$\begin{cases} \boldsymbol{EC_{Met}} = \dfrac{\left(\boldsymbol{f_u^h C_h}\right)\boldsymbol{E^{tr}}}{\boldsymbol{K_{m,1}}} \\[2ex] \boldsymbol{EC_{Inh}} = \dfrac{\left(\boldsymbol{f_u^h C_h}\right)\boldsymbol{E^{tr}}}{\boldsymbol{K_i}} \\[2ex] \boldsymbol{EC_{MBI}} = \dfrac{\left(\boldsymbol{f_u^h C_h}\right)\boldsymbol{E^{tr}}}{\boldsymbol{K_I}} \end{cases} \tag{S2.2}$$

where the constants$\boldsymbol{K_{m,1}} = \dfrac{\boldsymbol{k_{21}}+\boldsymbol{k_{cat}}}{\boldsymbol{k_{12}}}$ $\boldsymbol{K_i} = \dfrac{\boldsymbol{k_{31}}}{\boldsymbol{k_{13}}}$ and $\boldsymbol{K_I} = \dfrac{\boldsymbol{k_{41}}+\boldsymbol{k_{inact}}}{\boldsymbol{k_{14}}}$. This allows to simplify Eq (S2.1) further:

$$
\begin{aligned}
\frac{d\boldsymbol{C_h}}{dt} &= \alpha_{H\to B}(x)\left[(\boldsymbol{P}+\boldsymbol{\rho_{in}}).\boldsymbol{f_u^b}.\boldsymbol{C_b} - (\boldsymbol{P}+\boldsymbol{\rho_{out}}).\boldsymbol{f_u^h}.\boldsymbol{C_h}\right] \\
&\quad - (\boldsymbol{k_{cat}}.\boldsymbol{EC_{Met}})\,\mathbb{1}_{\boldsymbol{n_E}} \\
&\quad - (\boldsymbol{k_{inact}}.\boldsymbol{EC_{MBI}})\,\mathbb{1}_{\boldsymbol{n_E}} \\
&\quad - \frac{\boldsymbol{V_{max,2}}}{\boldsymbol{K_{m,2}}+\boldsymbol{f_u^h}.\boldsymbol{C_h}}.\boldsymbol{f_u^h}.\boldsymbol{C_h} \\
&= \alpha_{H\to B}(x)\left[(\boldsymbol{P}+\boldsymbol{\rho_{in}}).\boldsymbol{f_u^b}.\boldsymbol{C_b} - (\boldsymbol{P}+\boldsymbol{\rho_{out}}).\boldsymbol{f_u^h}.\boldsymbol{C_h}\right] \\
&\quad - \left[(\boldsymbol{k_{cat}}/\boldsymbol{K_{m,1}}+\boldsymbol{k_{inact}}/\boldsymbol{K_I})\,\boldsymbol{E}\right].\boldsymbol{f_u^h}.\boldsymbol{C_h} - \frac{\boldsymbol{V_{max,2}}}{\boldsymbol{K_{m,2}}+\boldsymbol{f_u^h}.\boldsymbol{C_h}}.\boldsymbol{f_u^h}.\boldsymbol{C_h}
\end{aligned}
\tag{S2.3}
$$

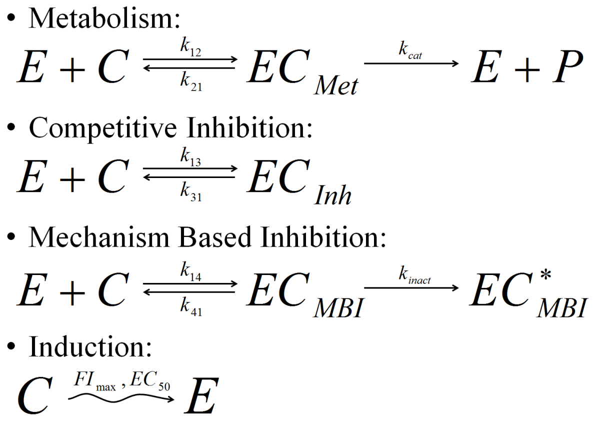

Figure S2.1: **Enzymatic reactions taking into account in the liver model.** Reversible inhibition: A drug binds to an enzyme which may result in its metabolism (but not necessarily) resulting in the temporary blockade or inhibition of the enzyme. Here only competitive inhibition will be studied, which assumes that each enzyme can interact with one drug at a time. Mechanism Based Inhibition (MBI): A drug inactivates an enzyme through direct interaction resulting in an inhibited metabolism of any drug metabolized by these enzymes. Induction: A drug induces the expression of one or more enzymes resulting in an induced metabolism of any drug metabolized by these enzymes. Note that the notations in this figure regarding the kinetic rate constants are used in the text.

S2.2 Enzymes

The remaining equations need to describe the enzyme kinetics of the drugs. In general the level of enzymes are assumed constant, but when MBI and/or

induction occur, the process is not immediate and therefore needs to be taken into consideration, which will also affect the metabolism of the drugs. Using classical kinetic equations and assuming that the enzyme induction is additive, one can set:

$$\begin{cases} \frac{d\boldsymbol{E}}{dt} = \boldsymbol{k_{deg}}. \left[\boldsymbol{E_0} + \left(\frac{\left(\boldsymbol{E_{max}} - \mathbb{1}_{\boldsymbol{n_C}} \boldsymbol{E_0}^{tr}\right)}{\boldsymbol{EC_{50}} + \left(\boldsymbol{f_u^h}.\boldsymbol{C_h}\right) \mathbb{1}_{\boldsymbol{n_E}}^{tr}} \right)^{tr} \left(\boldsymbol{f_u^h}.\boldsymbol{C_h}\right) - \boldsymbol{E_{Tot}} \right] \\ \qquad - \left[\boldsymbol{k_{12}}. \left(\left(\boldsymbol{f_u^h}.\boldsymbol{C_h}\right) \boldsymbol{E}^{tr}\right) - \boldsymbol{k_{21}}.\boldsymbol{EC_{Met}} - \boldsymbol{k_{cat}}.\boldsymbol{EC_{Met}}\right]^{tr} \mathbb{1}_{\boldsymbol{n_C}} \\ \qquad - \left[\boldsymbol{k_{13}}. \left(\left(\boldsymbol{f_u^h}.\boldsymbol{C_h}\right) \boldsymbol{E}^{tr}\right) - \boldsymbol{k_{31}}.\boldsymbol{EC_{Inh}}\right]^{tr} \mathbb{1}_{\boldsymbol{n_C}} \\ \qquad - \left[\boldsymbol{k_{14}}. \left(\left(\boldsymbol{f_u^h}.\boldsymbol{C_h}\right) \boldsymbol{E}^{tr}\right) - \boldsymbol{k_{41}}.\boldsymbol{EC_{MBI}}\right]^{tr} \mathbb{1}_{\boldsymbol{n_C}} \\ \frac{d\boldsymbol{EC_{Met}}}{dt} = \boldsymbol{k_{12}}. \left(\left(\boldsymbol{f_u^h}.\boldsymbol{C_h}\right) \boldsymbol{E}^{tr}\right) - \boldsymbol{k_{21}}.\boldsymbol{EC_{Met}} - \boldsymbol{k_{cat}}.\boldsymbol{EC_{Met}} \\ \frac{d\boldsymbol{EC_{Inh}}}{dt} = \boldsymbol{k_{13}}. \left(\left(\boldsymbol{f_u^h}.\boldsymbol{C_h}\right) \boldsymbol{E}^{tr}\right) - \boldsymbol{k_{31}}.\boldsymbol{EC_{Inh}} \\ \frac{d\boldsymbol{EC_{MBI}}}{dt} = \boldsymbol{k_{14}}. \left(\left(\boldsymbol{f_u^h}.\boldsymbol{C_h}\right) \boldsymbol{E}^{tr}\right) - \boldsymbol{k_{41}}.\boldsymbol{EC_{MBI}} - \boldsymbol{k_{inact}}.\boldsymbol{EC_{MBI}} \\ \boldsymbol{E_{Tot}} = \boldsymbol{E} + \left[\boldsymbol{EC_{Met}} + \boldsymbol{EC_{Inh}} + \boldsymbol{EC_{MBI}}\right]^{tr} \mathbb{1}_{\boldsymbol{n_C}} \end{cases} \tag{S2.4}$$

As above, assuming that the equilibrium between the drugs and enzyme complex is quickly reached, one can show that:

$$\begin{cases} \frac{d\boldsymbol{E}}{dt} \approx \frac{d\boldsymbol{E_{Tot}}}{dt} = \boldsymbol{k_{deg}}. \left[\boldsymbol{E_0} + \left(\frac{\left(\boldsymbol{E_{max}} - \mathbb{1}_{\boldsymbol{n_C}} \boldsymbol{E_0}^{tr}\right)}{\boldsymbol{EC_{50}} + \left(\boldsymbol{f_u^h}.\boldsymbol{C_h}\right) \mathbb{1}_{\boldsymbol{n_E}}^{tr}} \right)^{tr} \left(\boldsymbol{f_u^h}.\boldsymbol{C_h}\right) - \boldsymbol{E_{Tot}} \right] \\ \qquad - \left[\boldsymbol{k_{inact}}.\boldsymbol{EC_{MBI}}\right]^{tr} \mathbb{1}_{\boldsymbol{n_C}} \\ \frac{d\boldsymbol{EC_{Met}}}{dt} = \frac{d\boldsymbol{EC_{Inh}}}{dt} = \frac{d\boldsymbol{EC_{MBI}}}{dt} \approx 0 \\ \boldsymbol{E_{Tot}} = \boldsymbol{E} + \left[\boldsymbol{EC_{Met}} + \boldsymbol{EC_{Inh}} + \boldsymbol{EC_{MBI}}\right]^{tr} \mathbb{1}_{\boldsymbol{n_C}} \end{cases} \tag{S2.5}$$

The equation can be further simplified using Eq (S2.2), normalizing the enzyme level by its normal level $\overline{\boldsymbol{E}}_{\boldsymbol{Tot}} = \boldsymbol{E}/\boldsymbol{E_0}$and setting $\boldsymbol{FI_{max}} = \boldsymbol{E_{max}}/\boldsymbol{E_0}$, which gives:

$$
\begin{cases}
\dfrac{d\overline{\boldsymbol{E}}_{\boldsymbol{Tot}}}{dt} = \boldsymbol{k_{deg}}. \left[1 + \left(\dfrac{(\boldsymbol{FI_{max}} - 1)}{\boldsymbol{EC_{50}} + (\boldsymbol{f_u^h}.\boldsymbol{C_h})\, \mathbb{1}_{\boldsymbol{n_E}}^{tr}} \right)^{tr} (\boldsymbol{f_u^h}.\boldsymbol{C_h}) \right. \\
\qquad\qquad \left. - \overline{\boldsymbol{E}}_{\boldsymbol{Tot}}. \left(1 + \dfrac{\dfrac{1}{\boldsymbol{k_{deg}}}. \left(\frac{\boldsymbol{k_{inact}}}{\boldsymbol{K_I}} \right)^{tr} (\boldsymbol{f_u^h}.\boldsymbol{C_h})}{1 + \left(\frac{1}{\boldsymbol{K_{m,1}}} + \frac{1}{\boldsymbol{K_i}} + \frac{1}{\boldsymbol{K_I}} \right)^{tr} (\boldsymbol{f_u^h}.\boldsymbol{C_h})} \right) \right] \\
\overline{\boldsymbol{E}} = \dfrac{\overline{\boldsymbol{E}}_{\boldsymbol{Tot}}}{1 + \left(\frac{1}{\boldsymbol{K_{m,1}}} + \frac{1}{\boldsymbol{K_i}} + \frac{1}{\boldsymbol{K_I}} \right)^{tr} (\boldsymbol{f_u^h}.\boldsymbol{C_h})}
\end{cases}
\tag{S2.6}
$$
