# Supplementary material for: A quantitative systems pharmacology approach, incorporating a novel liver model, for predicting pharmacokinetic drug-drug interactions
Source: PLoS One. 2017 Sep 14;12(9):e0183794. doi: 10.1371/journal.pone.0183794 (PMC5598964; doi:10.1371/journal.pone.0183794)
Supplement: S3 Appendix — The two definitions of the parameter Qg by Yang et al. [17] and Hisaka et al. [16] are presented. (PDF) [file pone.0183794.s003.pdf]

S3 Appendix: The Hybride Parameter Q_g

Q_g is a hybrid parameter introduced by Yang *et al.* [1] to take into account the permeability and blood flow from the enterocytes to the portal vein. Yang suggested to fix this parameters as:

$$Q_g^{Yang} = \frac{CL_{perm} Q_v}{CL_{perm} + Q_v} \tag{S3.1}$$

where Q_v is the villous blood flow and fixed at $18\,\mathrm{L/h}$ and CL_{perm} is the permeability through the enterocytes and is calculated from $CL_{perm} = S_{Entrocytes} * P_{eff}$, where $S_{Entrocytes}$ is equal to $0.66\,\mathrm{m}^2$ [1] and P_{eff} is supposed to be similar to the permeability in the hepatocytes.

However, Hisaka *et al.* [2] introduced a mechanistic model which takes into account bi-directional permeability across the enterocytes and all parameters are expressed as a function of free concentration, a different formula is proposed and will be compared to Eq (S3.1). Assuming negligible permeability from basolateral to apical, Hisaka's equation is given by:

$$Q_g^{Hisaka} = \frac{CL_{perm} \frac{Q_v}{f_u^b}}{CL_{perm} + \frac{Q_v}{f_u^b}} \tag{S3.2}$$

Further explanation on how Eq (S3.2) is derived can be found in S4 Appendix. Note that if Eq (S3.2) is multiplied by f_u^b then $CL_{perm}^{Yang} = f_u^b CL_{perm}^{Hisaka}$ within Yang's formula and:

$$Q_g^{Yang} = f_u^b Q_g^{Hisaka} \tag{S3.3}$$

For the simulation, Q_g refers to the definition given by Hisaka *et al.*

References

[1] J. Yang, M. Jamei, K. R. Yeo, G. T. Tucker, A. Rostami-Hodjegan, Prediction of intestinal first-pass drug metabolism., Current Drug Metabolism 8 (7) (2007) 676–684. doi:10.2174/138920007782109733.

[2] A. Hisaka, Y. Ohno, T. Yamamoto, H. Suzuki, Theoretical Considerations on Quantitative Prediction of Drug-Drug Interactions., Drug Metabolism and Pharmacokinetics 25 (1) (2010) 48–61. doi:10.2133/dmpk.25.48. URL http://linkinghub.elsevier.com/retrieve/pii/S1347436715300057
