# Supplementary material for: A quantitative systems pharmacology approach, incorporating a novel liver model, for predicting pharmacokinetic drug-drug interactions
Source: PLoS One. 2017 Sep 14;12(9):e0183794. doi: 10.1371/journal.pone.0183794 (PMC5598964; doi:10.1371/journal.pone.0183794)
Supplement: S4 Appendix — Description of how Qg is deduced by Hisaka et al. [16]. (PDF) [file pone.0183794.s004.pdf]

S4 Appendix: Hisaka Equation for Q_g

In [1], Yang *et al.* defined Q_g as a hybrid parameter taking into account the blood flow from the enterocytes and the permeability through the gut wall. They suggested to take Q_g as:

$$Q_g^{Yang} = \frac{CL_{perm} Q_v}{CL_{perm} + Q_v} \tag{S4.1}$$

where Q_v is the villous blood flow and CL_{perm} is the permeability through the enterocytes. This equation expresses the permeability as a function of the total gut wall permeability. In order to correct the equation for free concentration, as described by Hisaka *et al.* [2], Eq (S3.2) was proposed. Let's assume that the gut wall is composed of two sub-compartments:

1. The enterocytes compartment (E-compartment), where the nutrients and drugs are absorbed and the intestinal metabolism occurs. The dynamic of this sub-compartment is described by:

$$V_E \frac{dC_E}{dt} = k_a F_a D e^{-k_a t} - CL_{perm}\left(f_u^E C_E - f_u^M C_M\right) - CL_{int}^{u,E} f_u^E C_E \tag{S4.2}$$

2. The muscle compartment (M-compartment), where the blood flows and the exchange occurs. The dynamic of this sub-compartment is described by:

$$V_M \frac{dC_M}{dt} = CL_{perm}\left(f_u^E C_E - f_u^M C_M\right) + Q_v\left(C_{AB} - \frac{C_M R_{BP}}{K_{p,M}}\right) \tag{S4.3}$$

Assuming that the arterial blood concentration is negligible than the concentration in the muscle, the equation can be simplified to:

$$V_M \frac{dC_M}{dt} = CL_{perm}\left(f_u^E C_E - f_u^M C_M\right) - Q_v \frac{C_M R_{BP}}{K_{p,M}} \tag{S4.4}$$

Assuming that the M-compartment is at quasi-steady-state, C_M can be expressed as a function of C_E by:

$$C_M = \frac{CL_{perm} f_u^E C_E}{CL_{perm} f_u^M + \frac{Q_v R_{BP}}{K_{p,M}}} \tag{S4.5}$$

Then the flux to the blood can be expressed as a function of C_E:

$$Q_v \frac{C_M R_{BP}}{K_{p,M}} = \frac{CL_{perm} \frac{Q_v R_{BP}}{K_{p,M}}}{CL_{perm} f_u^M + \frac{Q_v R_{BP}}{K_{p,M}}} f_u^E C_E = Q_g f_u^E C_E \tag{S4.6}$$

Finally assuming that $K_{p,M} = \frac{f_u}{f_u^M}$ and $R_{BP} = \frac{f_u}{f_u^b}$, Eq (S3.2) is found:

$$Q_g^{Hisaka} = \frac{CL_{perm} \frac{Q_v}{f_u^b}}{CL_{perm} + \frac{Q_v}{f_u^b}} \tag{S4.7}$$

If the arterial blood concentration is not negligible compared to the the gut wall concentration then Q_g should be corrected. Similarly, if there is active transport into or out of the enterocytes, then this should be integrated into the model.

References

[1] J. Yang, M. Jamei, K. R. Yeo, G. T. Tucker, A. Rostami-Hodjegan, Prediction of intestinal first-pass drug metabolism., Current Drug Metabolism 8 (7) (2007) 676–684. doi:10.2174/138920007782109733.

[2] A. Hisaka, Y. Ohno, T. Yamamoto, H. Suzuki, Theoretical Considerations on Quantitative Prediction of Drug-Drug Interactions., Drug Metabolism and Pharmacokinetics 25 (1) (2010) 48–61. doi:10.2133/dmpk.25.48.
URL http://linkinghub.elsevier.com/retrieve/pii/S1347436715300057
