# Supplementary material for: A quantitative systems pharmacology approach, incorporating a novel liver model, for predicting pharmacokinetic drug-drug interactions
Source: PLoS One. 2017 Sep 14;12(9):e0183794. doi: 10.1371/journal.pone.0183794 (PMC5598964; doi:10.1371/journal.pone.0183794)
Supplement: S5 Appendix — Description of how the partition coefficient of a PK compartment composed of different tissues is calculated. (PDF) [file pone.0183794.s005.pdf]

S5 Appendix: Average Partition Coefficient

In order to reduce the number of compartments, all organs and tissues not involved in the absorption, distribution, metabolism or elimination were merged into one compartment called the Rest of the Body compartment (RB-compartment). This compartment requires, as well, a partition coefficient $K_{p,RB}$. In Table 5, $K_{p,RB}$ was expressed as:

$$K_{p,RB} = \frac{\sum_{i=1}^{n} V_i K_{p,i}}{\sum_{i=1}^{n} V_i} \tag{S5.1}$$

where $K_{p,i}$ and V_i are the partition coefficient and volume of the merged compartments.

In this section, a proof of Eq (S5.1) is proposed when quasi-steady-state is reached between the compartments and blood. The dynamic of each compartment is given by:

$$V_i \frac{dC_i}{dt} = Q_i \left(C_{AB} - \frac{C_i R_{BP}}{K_{p,i}} \right) \tag{S5.2}$$

By adding all the compartment, one can show:

$$V_T \frac{dC_T}{dt} = \sum_{i=1}^{n} V_i \frac{dC_i}{dt} = Q_T C_{AB} - R_{BP} \sum_{i=1}^{n} Q_i \frac{C_i}{K_{p,i}} \tag{S5.3}$$

where $C_T = \frac{\sum_{i=1}^{n} V_i C_i}{V_T}$, $V_T = \sum_{i=1}^{n} V_i$ and $Q_T = \sum_{i=1}^{n} Q_i$. In other words, C_T, V_T and Q_T represent the average concentration, the volume and blood flow of the RB-compartment. When quasi-steady-state is reached one can define $K_{p,RB}$ as:

$$K_{p,RB} = R_{BP} \frac{C_T}{C_{AB}} \tag{S5.4}$$

Finally, by using the definition for C_T and the fact that each compartment i is at quasi-steady-state, one can show:

$$\begin{aligned} K_{p,RB} &= R_{BP} \frac{C_T}{C_{AB}} = R_{BP} \frac{\sum_{i=1}^{n} V_i C_i}{V_T C_{AB}} \\ &= R_{BP} \frac{\sum_{i=1}^{n} V_i \frac{C_{AB} K_{p,i}}{R_{BP}}}{V_T C_{AB}} = \frac{\sum_{i=1}^{n} V_i K_{p,i}}{\sum_{i=1}^{n} V_i} \end{aligned} \tag{S5.5}$$

Therefore Eq (S5.1) is proven.
