# Supplementary material for: A quantitative systems pharmacology approach, incorporating a novel liver model, for predicting pharmacokinetic drug-drug interactions
Source: PLoS One. 2017 Sep 14;12(9):e0183794. doi: 10.1371/journal.pone.0183794 (PMC5598964; doi:10.1371/journal.pone.0183794)
Supplement: S6 Appendix — A brief description of Object-Oriented Programming (OOP) and a detail description on the program to solve the PBPK model. (PDF) [file pone.0183794.s006.pdf]

S6 Appendix: Numerical Resolution

In this section, the key ideas of the numerical methodology used to resolve the PBPK model are presented. As the program as been written using a object oriented programming (OOP) approach, the notions used in OOP are introduced in this section, followed by the main architecture of the program and then the numerical implementation for each compartment. MATLAB® R2015b [1] was used to write the code used for the simulation. The code files are attached for more details.

S6.1 Introduction to Object-Oriented Programming

Following the theoretical presentation of the liver model, it is necessary to offer an approach to solve/simulate the PBPK model considering all compartments. In fact, the main challenge with PBPK models is to design a methodology which facilitates their resolution including all compartments (that may have different levels of description) and that anticipates future needs of more advanced model for a particular compartment (*e.g.*: mechanistic and physiological description of the kidneys or the blood-brain barrier). This would need to be done without having to re-write all the code to incorporate those changes. Those constrains are common in different area in engineering, physics, systems biology or even quantitative finance and the common approach to resolve such problems is to use object-oriented programming. Many programming languages permit to use an OOP approach, as Python, C++, Java or MATLAB®. All these languages have their advantages and disadvantages, but the ability to develop a code easily and quickly without having the need to install mathematical libraries was preferred over a fast programming language, therefore MATLAB® was preferred.

OOP is a programming approach based on the concept of objects and how they interact with each others, which is different than the classical procedural approach of programming that focuses on functions (see figure S6.1). The objects are organized into classes that define the common properties, or attributes (*e.g.*: engine type, maximum engine power and amount of fuel for a vehicle), and common methods. This describe actions/behaviors of the object that can depend or affect its properties (*e.g.*: A running engine decreases the fuel amount). The properties and the methods are encapsulated into different classes, preventing the final users from misusing the properties and giving the appropriate tools (*i.e.*: the methods) to interact with the different objects (see figure S6.2). Moreover, a class B can inherit the properties and methods from a class A, which allows the creation of "abstract" classes without re-describing the common properties and methods for the "daughter" classes (see figure S6.3). Beside, polymorphism is also another key feature of OOP, which allows different classes to have the same method (*i.e.*: name), but different implementations (see figure S6.4). Both inheritance and polymorphism are the key features, facilitating future development of more complex models. This is achieved by creating abstract classes with essential properties and methods, necessary to resolve PBPK models. In the next section, the classes created to resolve the

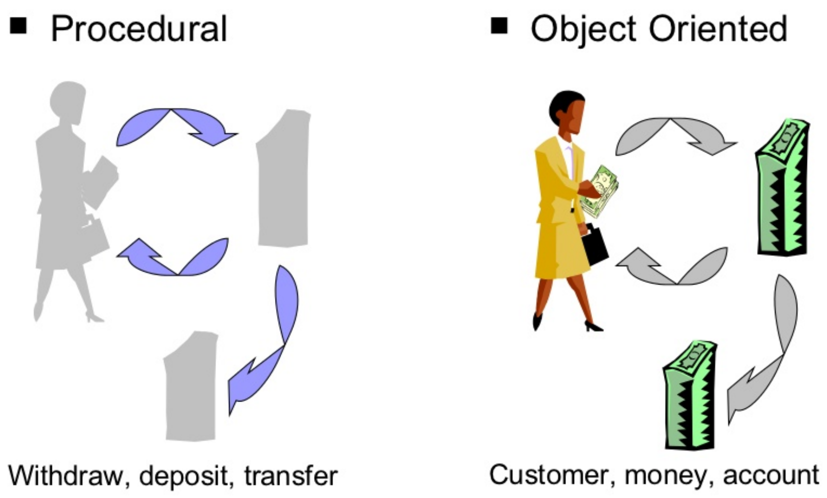

Figure S6.1: **Procedural vs. Object-Oriented programming.** Procedural programming focuses on functions where the action are described. Object-oriented programming focuses on the object which are defined by their properties and their behaviors.

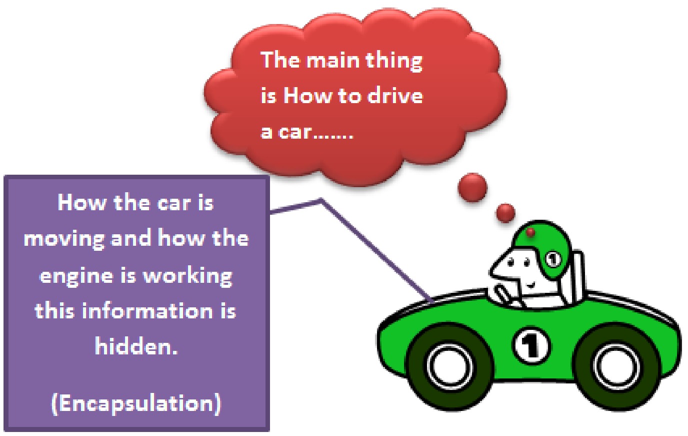

Figure S6.2: **Encapsulation of the properties and methods of an object.** While using a class, the user just to know the implementation of the methods. For example, to drive a car, one does not need to understand the mechanism of the engine but only the car controls.

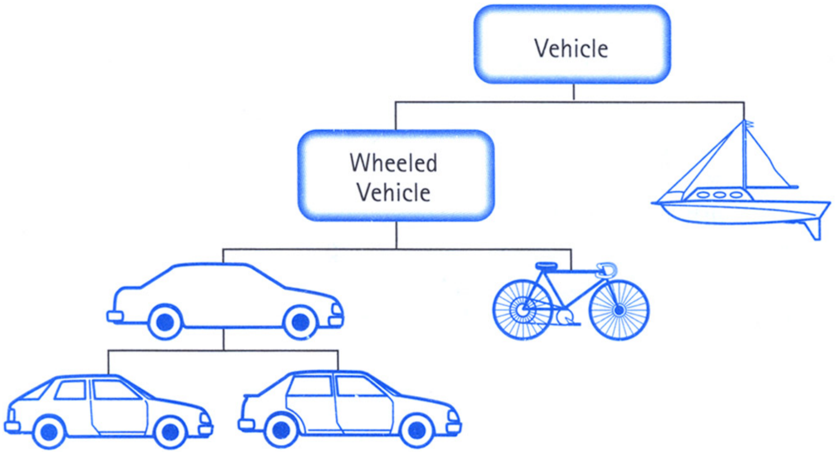

Figure S6.3: **Class inheritance.** A car, a bike or a boat are all vehicles than can move, turn, but with different type of way to move and number of wheel.

PBPK and the liver models are introduced.

S6.2 ODE, PBPK Compartment and PBPK system

Ordinary differential equations (ODEs) can be simply solved in MATLAB® by using one of the available solver, namely `ode15s` or `ode45`. In order to use these solvers, one needs to define a function $\boldsymbol{f}(t, \boldsymbol{Y})$, describing the dynamic of the system of interest such that $\boldsymbol{Y}$ represents the variables of a system and is solution of:

$$\frac{d\boldsymbol{Y}}{dt} = \boldsymbol{f}(t, \boldsymbol{Y}) \qquad \text{(S6.1)}$$

where $\boldsymbol{Y}$ and $\boldsymbol{f}$ are both column vectors of the size of the system of interest.

The main idea of solving a PBPK system using an OOP approach is to create an abstract class `OdeSystem` with an abstract methods that returns a function $\boldsymbol{f}$. The method will be inherited by all "daughter" classes with a different expression due to the polymorphism capability. The basic properties and methods of the class are represented in figure S6.5. From this abstract class, two main "daughter" classes are defined; `PbpkCpt` and `PbpkSystem` (see figure S6.6 and S6.7). The inheritance diagram of the different classes and an example of an `PbpkSystem` object are represented in figures S6.8 and S6.9, respectively.

As these two classes are defined, the creation of sub-classes representing compartments with one node (*e.g.*: `PbpkCpt_One` in figure S6.8) or more complex compartment as the gut or the liver model is facilitated. In each "daughter" class, the parameters of the compartment are defined as properties of the class. Therefore after defining them, there is no more need to manipulate them.

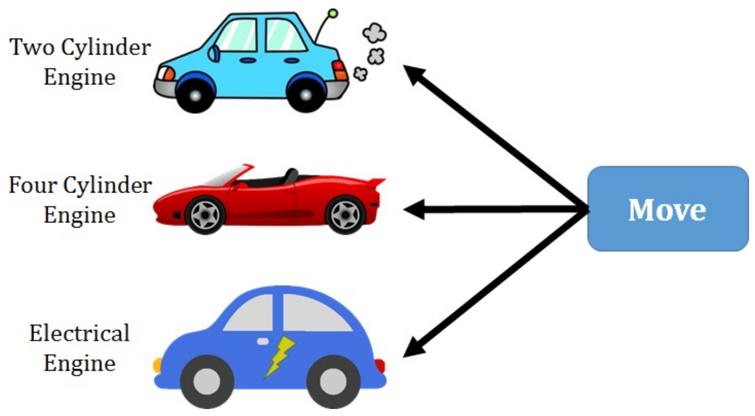

Figure S6.4: **Polymorphism.** Polymorphism is feature of OOP, which allows different classes to have the same method (*i.e.*: name), but different implementations. For example, all car have a pedal to make the car moving, but the engine could be different.

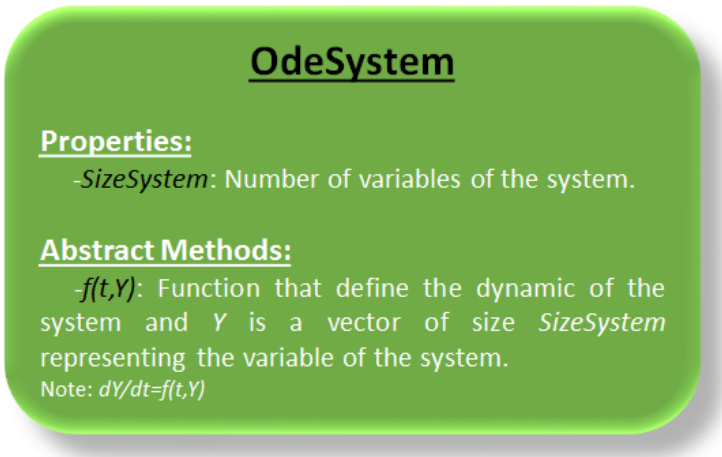

Figure S6.5: **Description of the class `OdeSystem`.**

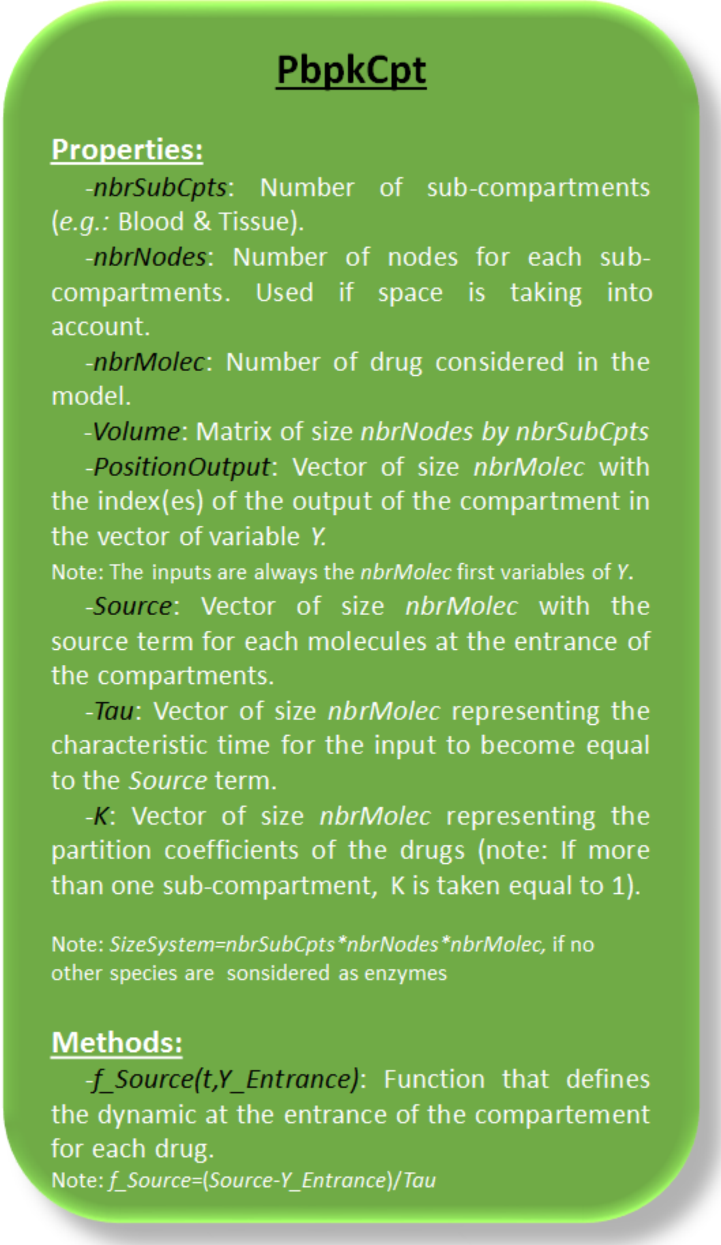

Figure S6.6: **Description of the class `PbpkCpt`.** `PbpkCpt` describes the main properties and methods of a PBPK compartment.

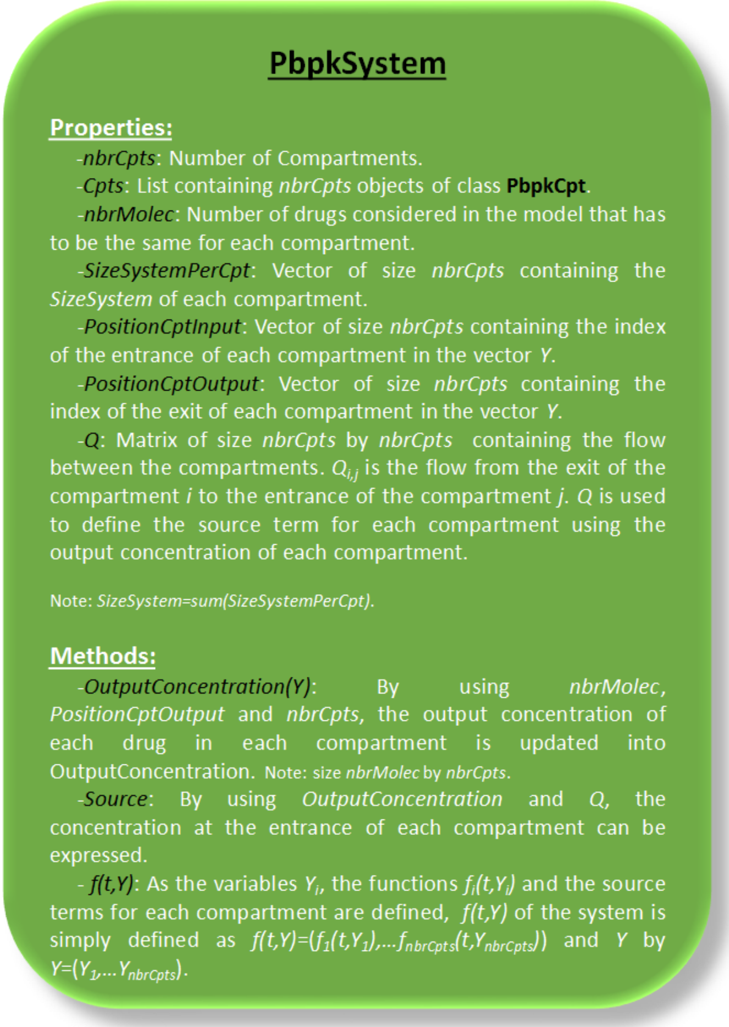

Figure S6.7: **Description of the class `PbpkSystem`.** `PbpkSystem` defines a PBPK system composed of objects of class `PbpkCpt` and has a matrix Q describing the flow between the different compartments. The function $\boldsymbol{f}$ and the variable $\boldsymbol{Y}$ of a PBPK system are simply the concatenation of the functions $\boldsymbol{f_k}$ and the variables $\boldsymbol{Y_k}$ of the n_{Cpt} compartment such as $\boldsymbol{f}^{tr} = \left(\boldsymbol{f_1}^{tr}, \boldsymbol{f_2}^{tr}, \ldots, \boldsymbol{f}^{tr}_{\boldsymbol{n_{Cpt}}}\right)$ and $\boldsymbol{Y}^{tr} = \left(\boldsymbol{Y_1}^{tr}, \boldsymbol{Y_2}^{tr}, \ldots, \boldsymbol{Y}^{tr}_{\boldsymbol{n_{Cpt}}}\right)$.

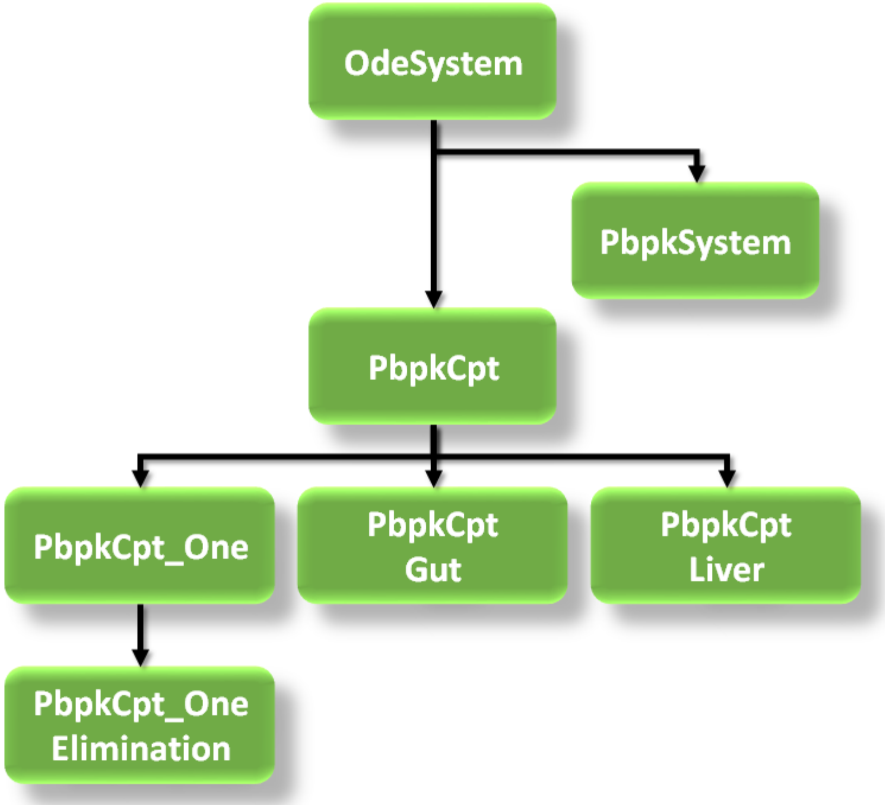

Figure S6.8: **Inheritance diagram of the different classes.** A “daughter” class inherits all properties and methods of its “mother” class. Therefore, there is no need to re-define them when creating the “daughter” class. Nevertheless, if a method needs to be implemented differently, it could be re-defined due to the polymorphism capability as for the function $\boldsymbol{f}$ in this case.

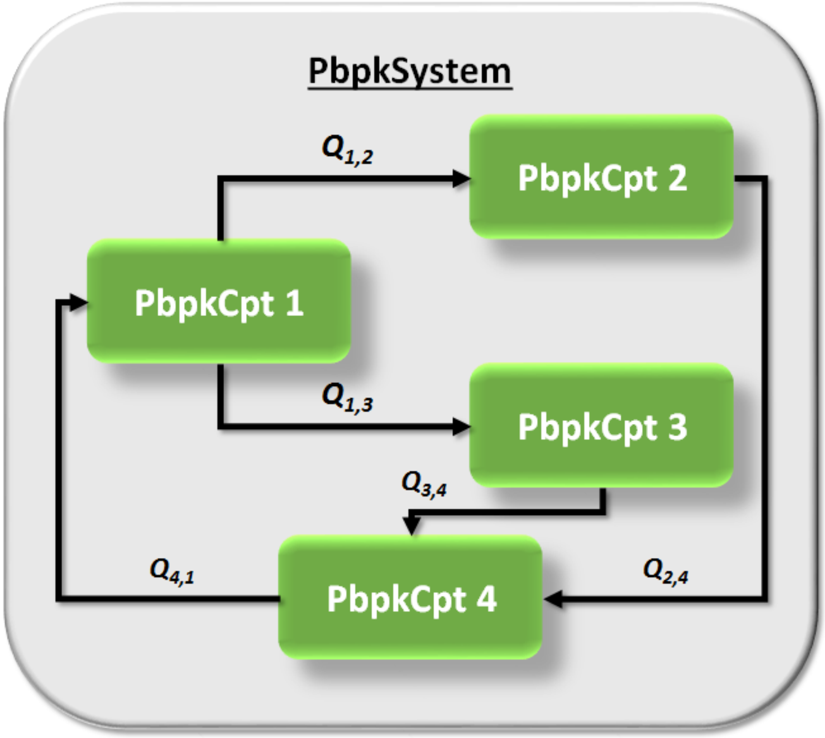

Figure S6.9: **Example of a `PbpkSystem` object composed of 4 `PbpkCpt` objects.**

S6.3 Simple Compartment

A simple compartment is a compartment that is homogeneous in space, therefore only one variable is necessary per compound. The classes `PbpkCpt_One_Elimination` and `PbpkCpt_One` are examples of simple compartment. For instance, when the elimination rate $\boldsymbol{k_{el}}$ is accounting for, the function $\boldsymbol{f_{sc}}$ is written as:

$$\boldsymbol{f_{sc}}\left(t,\boldsymbol{Y}\right)=\left(\boldsymbol{S}-\boldsymbol{Y_{sc}}./\boldsymbol{K_p}\right)/\tau-\boldsymbol{k_{el}}.\boldsymbol{Y_{sc}} \tag{S6.2}$$

where $\boldsymbol{Y_{sc}}$ represents the concentration of the compounds, $\boldsymbol{K_p}$ the partition coefficients of the compounds, $\tau = V/Q$ the characteristic time for the arterial blood to reach the compartment (V and Q are the volume and flow of the compartment) and $\boldsymbol{S}$ the source concentration of the compounds at the entrance of the compartment. The notations presented in the paper, which are similar to MATLAB® script, are used.

The arterial blood, venous blood, kidney, lung and RB compartments are simple compartment with only the kidney compartment having an elimination rate constant.

S6.4 Gut Compartment

The gut compartment is composed of two sub-compartments; portal vein and gut wall. The variables of the system are $\boldsymbol{C_{pv}}$, $\boldsymbol{C_g}$ and $\overline{\boldsymbol{E}}_{\boldsymbol{Tot,g}}$, which are the compound concentrations in the portal vein and in the gut walls and the enzyme levels in the gut wall, respectively. They are solutions of Eqs (15) and (16). The variable $\boldsymbol{Y_G}$ and the function $\boldsymbol{f_G}$ of the gut compartment are defined as:

$$\begin{cases}\boldsymbol{Y_G^{tr}}=\left(\boldsymbol{C_{pv}^{tr}},\boldsymbol{C_g^{tr}},\overline{\boldsymbol{E}}_{\boldsymbol{Tot,g}}^{tr}\right)\\ \boldsymbol{f_G^{tr}}\left(t,\boldsymbol{Y_G}\right)=\left(\boldsymbol{f_{pv}^{tr}}\left(t,\boldsymbol{C_{pv}},\boldsymbol{C_g}\right),\boldsymbol{f_{g,C}^{tr}}\left(t,\boldsymbol{C_g},\overline{\boldsymbol{E}}_{\boldsymbol{Tot,g}}\right),\boldsymbol{f_{g,E}^{tr}}\left(t,\boldsymbol{C_g},\overline{\boldsymbol{E}}_{\boldsymbol{Tot,g}}\right)\right)\end{cases} \tag{S6.3}$$

where:

$$
\begin{cases}
\boldsymbol{f_{pv}}\left(t,\boldsymbol{C_{pv}},\boldsymbol{C_g}\right) = \dfrac{Q_{pv}}{V_{pv}}\left(\boldsymbol{S_G}-\boldsymbol{C_{pv}}\right) + \dfrac{Q_g}{V_{pv}}\boldsymbol{f_u^g}.\boldsymbol{C_g} \\[2ex]
\boldsymbol{f_{g,C}}\left(t,\boldsymbol{C_g},\overline{\boldsymbol{E}}_{\boldsymbol{Tot,g}}\right) = \displaystyle\sum_{i=1}^{n_{Dose}} \dfrac{\boldsymbol{F_a}.\boldsymbol{D_i}.\boldsymbol{k_a}}{V_g}.\exp\left(-\boldsymbol{k_a}\left(t-T_i\right)\right)H\left(t-T_i\right) \\
\qquad - \left[\left(\boldsymbol{k_{cat}^g}/\boldsymbol{K_m^g}+\boldsymbol{k_{inact}^g}/\boldsymbol{K_I^g}\right)\boldsymbol{E_g}\right].\boldsymbol{f_u^g}.\boldsymbol{C_g} \\
\qquad - \dfrac{\boldsymbol{V_{max}^g}}{\boldsymbol{K_{m,2}^g}+\boldsymbol{f_u^g}.\boldsymbol{C_h}}.\boldsymbol{f_u^g}.\boldsymbol{C_g} - \dfrac{Q_g}{V_g}\boldsymbol{f_u^g}.\boldsymbol{C_g} \\[2ex]
\boldsymbol{f_{g,E}}\left(t,\boldsymbol{C_g},\overline{\boldsymbol{E}}_{\boldsymbol{Tot,g}}\right) = \boldsymbol{k_{deg}^g}.\left[1+\left(\dfrac{\left(\boldsymbol{FI_{max}^g}-1\right)}{\boldsymbol{EC_{50}^g}+\left(\boldsymbol{f_u^g}.\boldsymbol{C_g}\right)\mathbb{1}_{\boldsymbol{n_E}}^{tr}}\right)^{tr}\left(\boldsymbol{f_u^g}.\boldsymbol{C_g}\right)\right. \\
\qquad \left. -\overline{\boldsymbol{E}}_{\boldsymbol{Tot,g}}.\left(1+\dfrac{\frac{1}{\boldsymbol{k_{deg}^g}}.\left(\frac{\boldsymbol{k_{inact}^g}}{\boldsymbol{K_I^g}}\right)^{tr}\left(\boldsymbol{f_u^g}.\boldsymbol{C_g}\right)}{1+\left(\frac{1}{\boldsymbol{K_{m,1}^g}}+\frac{1}{\boldsymbol{K_i^g}}+\frac{1}{\boldsymbol{K_I^g}}\right)^{tr}\left(\boldsymbol{f_u^g}.\boldsymbol{C_g}\right)}\right)\right]
\end{cases}
\tag{S6.4}
$$

with $\boldsymbol{E_g}\left(\boldsymbol{C_g},\overline{\boldsymbol{E}}_{\boldsymbol{Tot,g}}\right) = \boldsymbol{E_{0,g}}.\dfrac{\overline{\boldsymbol{E}}_{\boldsymbol{Tot,g}}}{1+\left(\frac{1}{\boldsymbol{K_{m,1}^g}}+\frac{1}{\boldsymbol{K_i^g}}+\frac{1}{\boldsymbol{K_I^g}}\right)^{tr}\left(\boldsymbol{f_u^g}.\boldsymbol{C_g}\right)}$ and $\boldsymbol{S_G}$ the source concentration of the compounds at the portal vein entrance which is taken equal to arterial blood concentration $\boldsymbol{C_{AB}}$.

S6.5 Liver Compartment

Similarly to the gut compartment, the liver compartment is described by two sub-compartments; the sinusoids (blood capillaries) and the hepatocytes. The variables of the system are $\boldsymbol{C_b}$ for the compound concentrations in the sinusoid sub-compartment and $\boldsymbol{C_h}$ and $\overline{\boldsymbol{E}}_{\boldsymbol{Tot}}$ for the compound concentrations and enzyme levels in the hepatocytes sub-compartment. Unlike the gut compartment, space is taking into account, which requires an extra step to fully defines the variable $\boldsymbol{Y_L}$ and the function $\boldsymbol{f_L}$ of the liver compartment as the number of variables depends on the chosen spatial step. First, the variable $\boldsymbol{Y_L}$ will be defined in order to simplify the notations, followed by the description of the function $\boldsymbol{f_L}$.

One can set the spatial discretization; $x_1 = 0 < x_2 < \ldots, < x_{m-1} < x_m = \mathcal{L}_n$, where m is the number of spatial points and $\mathcal{L}_n$ is the length of all sinusoid levels (*i.e.* $= \sum_{k=1}^{n} L_k$; see Fig 1). Therefore, $\boldsymbol{C_b}$, $\boldsymbol{C_h}$ and $\overline{\boldsymbol{E}}_{\boldsymbol{Tot}}$ are estimated in m points. To simplify the notation, one can define $\boldsymbol{C_b}$ and $\boldsymbol{C_h}$ as matrices of size $n_C \times m$ and $\overline{\boldsymbol{E}}_{\boldsymbol{Tot}}$ as a matrix of size $n_E \times m$ where n_C and n_E are the number of compounds and enzymes considered, such as:

$$\begin{cases} \boldsymbol{C_b} = \left(\boldsymbol{C_b}\left(t,x_1\right), \boldsymbol{C_b}\left(t,x_2\right), \ldots, \boldsymbol{C_b}\left(t,x_m\right)\right) = \left(\boldsymbol{C_{b,1}}, \boldsymbol{C_{b,2}}, \ldots, \boldsymbol{C_{b,m}}\right) \\ \\ \boldsymbol{C_h} = \left(\boldsymbol{C_h}\left(t,x_1\right), \boldsymbol{C_h}\left(t,x_2\right), \ldots, \boldsymbol{C_h}\left(t,x_m\right)\right) = \left(\boldsymbol{C_{h,1}}, \boldsymbol{C_{h,2}}, \ldots, \boldsymbol{C_{h,m}}\right) \\ \\ \overline{\boldsymbol{E}}_{\boldsymbol{Tot}} = \left(\overline{\boldsymbol{E}}_{\boldsymbol{Tot}}\left(t,x_1\right), \overline{\boldsymbol{E}}_{\boldsymbol{Tot}}\left(t,x_2\right), \ldots, \overline{\boldsymbol{E}}_{\boldsymbol{Tot}}\left(t,x_m\right)\right) \\ \qquad = \left(\overline{\boldsymbol{E}}_{\boldsymbol{Tot,1}}, \overline{\boldsymbol{E}}_{\boldsymbol{Tot,2}}, \ldots, \overline{\boldsymbol{E}}_{\boldsymbol{Tot,m}}\right) \end{cases} \tag{S6.5}$$

These notations are useful for matrix operations. But $\boldsymbol{Y_L}$ is a column vector and to relate those variables to $\boldsymbol{Y_L}$, the matrices $\boldsymbol{C_b}$, $\boldsymbol{C_h}$ and $\overline{\boldsymbol{E}}_{\boldsymbol{Tot}}$ need to be converted into column vector. The vectorization is done in MATLAB® by the operation `Vec_M=M(:);`, where $\boldsymbol{M}$ is a matrix. In this section, this operation is noted $\overrightarrow{\boldsymbol{M}}$ such as:

$$\overrightarrow{\boldsymbol{M}}^{tr} = \left(\boldsymbol{M_1}^{tr}, \boldsymbol{M_2}^{tr}, \ldots, \boldsymbol{M_m}^{tr}\right) \tag{S6.6}$$

Therefore, the vectorization of $\boldsymbol{C_b}$, $\boldsymbol{C_h}$ and $\overline{\boldsymbol{E}}_{\boldsymbol{Tot}}$ are:

$$\begin{cases} \overrightarrow{\boldsymbol{C_b}}^{tr} = \left(\boldsymbol{C_{b,1}}^{tr}, \boldsymbol{C_{b,2}}^{tr}, \ldots, \boldsymbol{C_{b,m}}^{tr}\right) \\ \\ \overrightarrow{\boldsymbol{C_h}}^{tr} = \left(\boldsymbol{C_{h,1}}^{tr}, \boldsymbol{C_{h,2}}^{tr}, \ldots, \boldsymbol{C_{h,m}}^{tr}\right) \\ \\ \overrightarrow{\boldsymbol{E_{Tot}}}^{tr} = \left(\overline{\boldsymbol{E}}_{\boldsymbol{Tot,1}}, \overline{\boldsymbol{E}}_{\boldsymbol{Tot,2}}, \ldots, \overline{\boldsymbol{E}}_{\boldsymbol{Tot,m}}\right) \end{cases} \tag{S6.7}$$

And finally $\boldsymbol{Y_L}$ is simply defined the concatenation of $\overrightarrow{\boldsymbol{C_b}}$, $\overrightarrow{\boldsymbol{C_h}}$ and $\overrightarrow{\boldsymbol{E_{Tot}}}$, such as:

$$\boldsymbol{Y_L}^{tr} = \left(\overrightarrow{\boldsymbol{C_b}}^{tr}, \overrightarrow{\boldsymbol{C_h}}^{tr}, \overrightarrow{\boldsymbol{E_{Tot}}}^{tr}\right) \tag{S6.8}$$

Now that the spatial discretization and $\boldsymbol{Y_L}$ have been defined, the function $\boldsymbol{f_L}$ can be expressed. Similarly to $\boldsymbol{Y_L}$, $\boldsymbol{f_L}$ is the concatenation of three functions $\boldsymbol{f_b}\left(t, \boldsymbol{C_b}, \boldsymbol{C_h}\right)$, $\boldsymbol{f_{h,C}}\left(t, \boldsymbol{C_b}, \boldsymbol{C_h}, \overline{\boldsymbol{E}}_{\boldsymbol{Tot}}\right)$ and $\boldsymbol{f_{h,E}}\left(t, \boldsymbol{C_h}, \overline{\boldsymbol{E}}_{\boldsymbol{Tot}}\right)$ that describes the dynamic of $\boldsymbol{C_b}$, $\boldsymbol{C_h}$ and $\overline{\boldsymbol{E}}_{\boldsymbol{Tot}}$, respectively, such as:

$$\begin{aligned} \boldsymbol{f_L}^{tr}\left(t, \boldsymbol{Y_L}\right) &= \left(\boldsymbol{f_b}^{tr}\left(t, \boldsymbol{C_b}, \boldsymbol{C_h}\right), \boldsymbol{f_{h,C}}^{tr}\left(t, \boldsymbol{C_b}, \boldsymbol{C_h}, \overline{\boldsymbol{E}}_{\boldsymbol{Tot}}\right), \boldsymbol{f}^{tr}_{h,\boldsymbol{E}}\left(t, \boldsymbol{C_h}, \overline{\boldsymbol{E}}_{\boldsymbol{Tot}}\right)\right) \\ &= \left(\overrightarrow{\boldsymbol{F_b}}^{tr}\left(t, \boldsymbol{C_b}, \boldsymbol{C_h}\right), \overrightarrow{\boldsymbol{F_{h,C}}}^{tr}\left(t, \boldsymbol{C_b}, \boldsymbol{C_h}, \overline{\boldsymbol{E}}_{\boldsymbol{Tot}}\right), \overrightarrow{\boldsymbol{F_{h,E}}}^{tr}\left(t, \boldsymbol{C_h}, \overline{\boldsymbol{E}}_{\boldsymbol{Tot}}\right)\right) \end{aligned} \tag{S6.9}$$

where $\boldsymbol{F_b}$, $\boldsymbol{F_{h,C}}$ and $\boldsymbol{F_{h,E}}$ are the matrix forms of $\boldsymbol{f_b}$, $\boldsymbol{f_{h,C}}$ and $\boldsymbol{f_{h,E}}$, respectively. $\boldsymbol{F_b}$ is expressed using Eq (1), which is a partial differential equation

(PDE). The simple scheme for a conservation equation is a backward Euler scheme, which gives:

$$\begin{cases} \boldsymbol{F_{b,1}} = -\alpha_{BH}(x_1)\left[(\boldsymbol{P}+\boldsymbol{\rho_{in}}).\boldsymbol{f_u^b}.\boldsymbol{C_{b,1}} - (\boldsymbol{P}+\boldsymbol{\rho_{out}}).\boldsymbol{f_u^h}.\boldsymbol{C_{h,1}}\right] \\ \qquad - \dfrac{1}{\tau_L}(\boldsymbol{S_L} - \boldsymbol{C_{b,1}}) \\ \boldsymbol{F_{b,k}} = -\alpha_{BH}(x_k)\left[(\boldsymbol{P}+\boldsymbol{\rho_{in}}).\boldsymbol{f_u^b}.\boldsymbol{C_{b,k}} - (\boldsymbol{P}+\boldsymbol{\rho_{out}}).\boldsymbol{f_u^h}.\boldsymbol{C_{h,k}}\right] \quad \forall k \in [\![2:m]\!] \\ \qquad - v(x_k)\dfrac{\boldsymbol{C_{b,k}} - \boldsymbol{C_{b,k-1}}}{x_k - x_{k-1}} \end{cases} \tag{S6.10}$$

where τ_L is the characteristic time for the arterial blood to reach the entrance of the lobule and is taken equal to $\frac{x_2-x_1}{v(x_1)}$. $\boldsymbol{S_L}$ represents the source concentration of the compounds at the entrance of the lobule. $\boldsymbol{F_{h,C}}$ and $\boldsymbol{F_{h,E}}$ are expressed using Eqs (7) and (9), such as:

$$\begin{cases} \boldsymbol{F_{h,C,k}} = \alpha_{HB}(x_k)\left[(\boldsymbol{P}+\boldsymbol{\rho_{in}}).\boldsymbol{f_u^b}.\boldsymbol{C_{b,k}} - (\boldsymbol{P}+\boldsymbol{\rho_{out}}).\boldsymbol{f_u^h}.\boldsymbol{C_{h,k}}\right] & \forall k \in [\![1:m]\!] \\ \qquad - \left[(\boldsymbol{k_{cat}}/\boldsymbol{K_{m,1}} + \boldsymbol{k_{inact}}/\boldsymbol{K_I})\,\boldsymbol{E_k}\right].\boldsymbol{f_u^h}.\boldsymbol{C_{h,k}} \\ \qquad - \dfrac{\boldsymbol{V_{max}}}{\boldsymbol{K_{m,2}} + \boldsymbol{f_u^h}.\boldsymbol{C_{h,k}}}.\boldsymbol{f_u^h}.\boldsymbol{C_{h,k}} \\ \boldsymbol{F_{h,E,k}} = \boldsymbol{k_{deg}}.\left[1 + \left(\dfrac{(\boldsymbol{FI_{max}} - 1)}{\boldsymbol{EC_{50}} + \left(\boldsymbol{f_u^h}.\boldsymbol{C_{h,k}}\right)\mathbb{1}_{\boldsymbol{n_E}}^{tr}}\right)^{tr}\left(\boldsymbol{f_u^h}.\boldsymbol{C_{h,k}}\right)\right. & \forall k \in [\![1:m]\!] \\ \qquad \left. -\overline{\boldsymbol{E}}_{\boldsymbol{Tot,k}}.\left(1 + \dfrac{\frac{1}{\boldsymbol{k_{deg}}}.\left(\frac{\boldsymbol{k_{inact}}}{\boldsymbol{K_I}}\right)^{tr}\left(\boldsymbol{f_u^h}.\boldsymbol{C_{h,k}}\right)}{1 + \left(\frac{1}{\boldsymbol{K_{m,1}}} + \frac{1}{\boldsymbol{K_i}} + \frac{1}{\boldsymbol{K_I}}\right)^{tr}\left(\boldsymbol{f_u^h}.\boldsymbol{C_{h,k}}\right)}\right)\right] \end{cases} \tag{S6.11}$$

with $\boldsymbol{E_k} = \boldsymbol{E}\left(\boldsymbol{C_{h,k}}, \overline{\boldsymbol{E}}_{\boldsymbol{Tot,k}}\right) = \boldsymbol{E_0}.\dfrac{\overline{\boldsymbol{E}}_{\boldsymbol{Tot,k}}}{1 + \left(\frac{1}{\boldsymbol{K_{m,1}}} + \frac{1}{\boldsymbol{K_i}} + \frac{1}{\boldsymbol{K_I}}\right)^{tr}\left(\boldsymbol{f_u^h}.\boldsymbol{C_{h,k}}\right)}$.

S6.6 Source Concentration

The source concentration $\boldsymbol{S} = (S_{i,j})_{\substack{1\le i\le n_C \\ 1\le j\le n_{Cpt}}}$, where $S_{i,j}$ is the source concentration of the drug i into the compartment j, for each compartment can be expressed as a function of the output concentration of all compartment $\boldsymbol{C_{Output}}$ and the flow between the compartments, noted $\boldsymbol{Q}$. $\boldsymbol{C_{Output}}$ is a matrix such as $\boldsymbol{C_{Output}} = (C_{Output,i,j})_{\substack{1\le i\le n_C \\ 1\le j\le n_{Cpt}}}$ and $C_{Output,i,j}$ is the output concentration of the drug i from the compartment j, corrected by the partition coefficient. $\boldsymbol{Q}$ is

a matrix such as $\boldsymbol{Q} = (Q_{i,j})_{\substack{1\leq i\leq n_{Cpt}\\ 1\leq j\leq n_{Cpt}}}$ and $Q_{i,j}$ is the flow from the compartment i to the compartment j. Therefore, $\boldsymbol{S}$ is simply given by:

$$\boldsymbol{S} = \frac{\boldsymbol{C_{Output}Q}}{\boldsymbol{Q_{In}}} \tag{S6.12}$$

where $\boldsymbol{Q_{In}} = \left(Q_{In,i} = \sum_{k=1}^{n_{Cpt}} Q_{k,i}\right)_{1\leq i\leq n_{Cpt}}$ represents the total flow into each compartment.

S6.7 Resolution

Now that the compartments have been described, the variable $\boldsymbol{Y}$ and the function $\boldsymbol{f}$ of the herein PBPK model is simply given by:

$$\begin{cases} \boldsymbol{Y}^{tr} = \left(\boldsymbol{Y}^{tr}_{\boldsymbol{AB}}, \boldsymbol{Y}^{tr}_{\boldsymbol{G}}, \boldsymbol{Y}^{tr}_{\boldsymbol{L}}, \boldsymbol{Y}^{tr}_{\boldsymbol{K}}, \boldsymbol{Y}^{tr}_{\boldsymbol{RB}}, \boldsymbol{Y}^{tr}_{\boldsymbol{VB}}, \boldsymbol{Y}^{tr}_{\boldsymbol{Lungs}}\right) \\ \boldsymbol{f}^{tr} = \left(\boldsymbol{f}^{tr}_{\boldsymbol{AB}}, \boldsymbol{f}^{tr}_{\boldsymbol{G}}, \boldsymbol{f}^{tr}_{\boldsymbol{L}}, \boldsymbol{f}^{tr}_{\boldsymbol{K}}, \boldsymbol{f}^{tr}_{\boldsymbol{RB}}, \boldsymbol{f}^{tr}_{\boldsymbol{VB}}, \boldsymbol{f}^{tr}_{\boldsymbol{Lungs}}\right) \end{cases} \tag{S6.13}$$

After defining the initial condition of the system $\boldsymbol{Y_0}$, the function $\boldsymbol{f}$ can simply be used with any MATLAB® to compute $\boldsymbol{Y}$ and obtain the pharmacokinetics and the enzyme levels of the different compounds. For the simulation presented in this thesis the solver `ode15s` was preferred, as it can solve stiff problem and adapt the time step for optimum resolution.

References

[1] Matlab, version 8.6 (R2015b), The MathWorks Inc., Natick, Massachusetts, 2015.
