# Supplementary material for: A quantitative systems pharmacology approach, incorporating a novel liver model, for predicting pharmacokinetic drug-drug interactions
Source: PLoS One. 2017 Sep 14;12(9):e0183794. doi: 10.1371/journal.pone.0183794 (PMC5598964; doi:10.1371/journal.pone.0183794)
Supplement: S1 Table — (PDF) [file pone.0183794.s007.pdf]

S1 Table: Average volume and blood flow for a $70\,\mathrm{kg}$ man for different tissues.

**Tissue**	Blood				
	RBC	*Plasma*	*Arterial*	*Venous*	*Total*
**Volume** (L)	2.2	3[1]	1.73[1, 2]	3.47[1, 2]	5.2[1]
**Blood Flow** (L/ min)	N/A	N/A	5.441[a]	5.441[a]	5.441[a]

**Tissue**	Adipose	Bone	Brain	Heart	Kidney
**Volume** (L)	10[1]	8.68[2]	1.45[1]	0.31[1]	0.28[1]
**Blood Flow** (L/ min)	0.26[1]	0.252[2]	0.7[1]	0.24[1]	1.24[1]

**Tissue**	Gut (Small Intestine)		Liver	Lungs	Muscle
	Gut Wall	*Portal Vein*			
**Volume** (L)	0.23[b]	0.34[c]	1.69[1]	1.17[1]	35[1]
**Blood Flow** (L/ min)	0.3 (Q_{Villi})	1.1[1]	1.45[1]	5.441[a]	0.75[1]

**Tissue**	Pancreas	Skin	Spleen	Stomach
**Volume** (L)	0.084[2]	7.8[1]	0.192[1]	0.154[2]
**Blood Flow** (L/ min)	0.133[2]	0.3[1]	0.077[1]	0.039[2]

[a] Deduced by summation of all other blood flows.

[b] Deduced from the small intestine volume of 575 mL [1] minus the volume of the blood content.

[c] Deduced from the total content of blood in the intestine (550 mL [3]) and assuming that the blood content was proportional to the small to large intestine ratio.

References

[1] B. Davies, T. Morris, Physiological Parameters In Laboratory Animals And Humans., Pharmaceutical Research 10 (7) (1993) 1093–1095. doi:10.1023/A:1018943613122.
URL http://www.springerlink.com/index/j302232361082w14.pdf

[2] S. A. Peters, Physiologically-Based Pharmacokinetic (PBPK) Modelling and Simulations, John Wiley & Sons, 2012.

[3] W. S. Snyder, M. J. Cook, E. S. Nasset, L. R. Karhausen, G. Parry Howells, I. H. Tipton, Report of the Task Group on Reference MAN., Tech. Rep. 23, ICRP (1975). arXiv:arXiv:1011.1669v3, doi:10.1007/s13398-014-0173-7.2.
