# Supplementary material for: A quantitative systems pharmacology approach, incorporating a novel liver model, for predicting pharmacokinetic drug-drug interactions
Source: PLoS One. 2017 Sep 14;12(9):e0183794. doi: 10.1371/journal.pone.0183794 (PMC5598964; doi:10.1371/journal.pone.0183794)
Supplement: S2 Table — (PDF) [file pone.0183794.s008.pdf]

S2 Table: Amounts and degradation rate constants for different CYP enzymes in the liver and the intestine.

Enzyme	Liver			Intestine		
	Amount nmol	*Fraction*	$\boldsymbol{k_{deg}}$ h^{-1}	*Amount* nmol	*Fraction*	$\boldsymbol{k^{g}_{deg}}$ h^{-1}
P-450 1A1	N/A	N/A	N/A	9.12	7.43%[1]	N/A
P-450 1A2	4037.39	12.21%[2]	N/A	N/A	N/A	N/A
P-450 2A6	1345.80	4.07%[2]	N/A	<0.37	<0.30%[1]	N/A
P-450 2B6	96.13	0.29%[2]	N/A	<0.37	<0.30%[1]	N/A
P-450 2C	5767.70	17.44%[2]	N/A	15.30	12.47%[1]	N/A
P-450 2D6	480.64	1.45%[2]	N/A	0.81	0.66%[1]	N/A
P-450 2E1	2114.82	6.40%[2]	N/A	<0.37	<0.30%[1]	N/A
P-450 2J2	N/A	N/A	N/A	1.47	1.19%[1]	N/A
P-450 3A4	9228.32[3]	27.91%[2]	0.0192 h^{-1}[4]	70.00[3]	57.03%[1]	0.0288 h^{-1}[4]
P-450 3A5	N/A	N/A	N/A	26.05	21.22%[1]	N/A
**Total P-450:**	**33068.15**			**122.74**		

References

[1] M. F. Paine, H. L. Hart, S. S. Ludington, R. L. Haining, A. E. Rettie, D. C. Zeldin, The Human Intestinal Cytochrome P450 "Pie"., Drug Metabolism and Disposition 34 (5) (2006) 880–886. doi:10.1124/dmd.105.008672.was. URL http://dmd.aspetjournals.org/cgi/doi/10.1124/dmd.105.008672

[2] T. Shimada, H. Yamazaki, M. Mimura, Y. Inui, F. P. Guengerich, Interindividual variations in human liver cytochrome P-450 enzymes involved in the oxidation of drugs, carcinogens and toxic chemicals: studies with liver microsomes of 30 Japanese and 30 Caucasians., The Journal of Pharmacology and Experimental Therapeutics 270 (1) (1994) 414–423.

[3] K. Rowland-Yeo, M. Jamei, J. Yang, G. T. Tucker, A. Rostami-Hodjegan, Physiologically based mechanistic modelling to predict complex drug-drug interactions involving simultaneous competitive and time-dependent enzyme inhibition by parent compound and its metabolite in both liver and gut - The effect of diltiazem on the time, European Journal of Pharmaceutical Sciences 39 (2010) 298–309. doi:10.1016/j.ejps.2009.12.002.

[4] O. A. Fahmi, S. Hurst, D. Plowchalk, J. Cook, F. Guo, K. Youdim, M. Dickins, A. Phipps, A. Darekar, R. Hyland, R. S. Obach, Comparison of different algorithms for predicting clinical drug-drug interactions, based on the use of CYP3A4 in vitro data: Predictions of compounds as precipitants of interaction., Drug Metabolism and Disposition 37 (2009) 1658–1666. doi:10.1124/dmd.108.026252.
