# Supplementary material for: A quantitative systems pharmacology approach, incorporating a novel liver model, for predicting pharmacokinetic drug-drug interactions
Source: PLoS One. 2017 Sep 14;12(9):e0183794. doi: 10.1371/journal.pone.0183794 (PMC5598964; doi:10.1371/journal.pone.0183794)
Supplement: S3 Table — Physico-chemical parameters used to calculate the partition coefficients. (PDF) [file pone.0183794.s009.pdf]

S3 Table: Physico-chemical properties of the drugs.

Drug	Nature	logP	$logD_{7.4}$	logP/D	$pK_{a,1}$	$pK_{a,2}$
Midazolam	Base	3.80[1]	3.78[1]	3.80	6.03[1]	N/A
Azithromycin	Base	2.44[a]	-1.23[a]	2.44	8.91[a]	N/A
Cimetidine	Base	-0.11[a]	-0.22[a]	-0.11	6.91[a]	N/A
Ethinyl Estradiol	Neutral	3.90[a]	3.90[a]	3.90	N/A	N/A
Rifampin	Zwitterion	2.05[1]	-0.46[1]	-0.46[1]	4.96[1]	7.30[1]

[a]Estimated with the software MarvinSketch, v15.7.6, 2015, ChemAxon (http://www.chemaxon.com) [2].

References

[1] A. P. Bento, A. Gaulton, A. Hersey, L. J. Bellis, J. Chambers, M. Davies, F. A. Kruger, Y. Light, L. Mak, S. McGlinchey, M. Nowotka, G. Papadatos, R. Santos, J. P. Overington, The ChEMBL bioactivity database: an update., Nucleic Acids Research 42 (D1) (2014) D1083–D1090. doi:10.1093/nar/gkt1031.
URL http://nar.oxfordjournals.org/lookup/doi/10.1093/nar/gkt1031

[2] MarvinSketch, v15.7.6, 2015, ChemAxon (http://www.chemaxon.com).
