# Supplementary material for: A quantitative systems pharmacology approach, incorporating a novel liver model, for predicting pharmacokinetic drug-drug interactions
Source: PLoS One. 2017 Sep 14;12(9):e0183794. doi: 10.1371/journal.pone.0183794 (PMC5598964; doi:10.1371/journal.pone.0183794)
Supplement: S4 Table — Tissue composition used to calculate the partitions coefficient. (PDF) [file pone.0183794.s010.pdf]

S4 Table: Composition of human tissue for different organs.

Tissue	f_{EW}	f_{IW}	f_{NL}	f_{NP}	Tissue-to-Plasma		$[AP]$
					Albumin	*Lipoprotein*	mg/g
Adipose	0.141[a]	0.039[a]	0.790[1]	0.002[1]	0.151[a]	0.068[a]	0.40[a]
Bone	0.098[a]	0.341[a]	0.074[1]	0.0005[1]	0.100[a]	0.050[a]	0.67[a]
Brain	0.092[a]	0.678[a]	0.051[1]	0.0565[1]	0.048[a]	0.041[a]	0.40[a]
Heart	0.313[a]	0.445[a]	0.0115[1]	0.0166[1]	0.157[a]	0.160[a]	3.07[a]
Muscle	0.091[a]	0.669[a]	0.0238[1]	0.0072[1]	0.271[a]	0.059[a]	2.49[a]
Pancreas	0.120[a]	0.664[a]	0.0723[1]	0.0188[1]	0.060[a]	0.060[a]	1.67[a]
Skin	0.623[a]	0.095[a]	0.0284[1]	0.0111[1]	0.277[a]	0.096[a]	1.32[a]
Spleen	0.208[a]	0.579[a]	0.0201[1]	0.0198[1]	0.097[a]	0.207[a]	2.81[a]
Stomach	0.292[a]	0.492[a]	0.0338[1]	0.0182[1]	0.158[a]	0.141[a]	2.41[a]
Kidney	0.283[a]	0.500[a]	0.0207[1]	0.0162[1]	0.130[a]	0.137[a]	2.48[a]
Lungs	0.348[a]	0.463[a]	0.003[1]	0.009[1]	0.212[a]	0.168[a]	0.50[a]
Gut	0.267[a]	0.451[a]	0.0487[1]	0.0163[1]	0.158[a]	0.141[a]	2.84[a]
Liver	0.165[a]	0.586[a]	0.0248[a]	0.0252[1]	0.086[a]	0.161[a]	5.09[a]
Plasma	0.945[a]	0	0.0035[a]	0.0022[a]	1	1	0.04[a]
Blood Cells	0	0.666[a]	0.0017[a]	0.0029[a]	0	0	0.44[a]

[a]Simcyp v12.2.

References

[1] S. A. Peters, Physiologically-Based Pharmacokinetic (PBPK) Modelling and Simulations, John Wiley & Sons, 2012.
